# Surface Reorganization of Transition Metal Dichalcogenide Nanoflowers for Efficient Electrochemical Coenzyme Regeneration

**DOI:** 10.1021/acsami.2c17483

**Published:** 2023-01-11

**Authors:** Nicholas Williams, Karley Hahn, Ryan Goodman, Xiaowen Chen, Jing Gu

**Affiliations:** † Department of Chemistry and Biochemistry, San Diego State University, 5500 Campanile Drive, San Diego, California92182, United States; ‡ Catalytic Carbon Transformation and Scale Up Center, National Renewable Energy Laboratory, 15013 Denver West Parkway, Golden, Colorado80401, United States

**Keywords:** electrochemical coenzyme generation, transition metal dichalcogenides, surface reorganization, catalyst activation

## Abstract

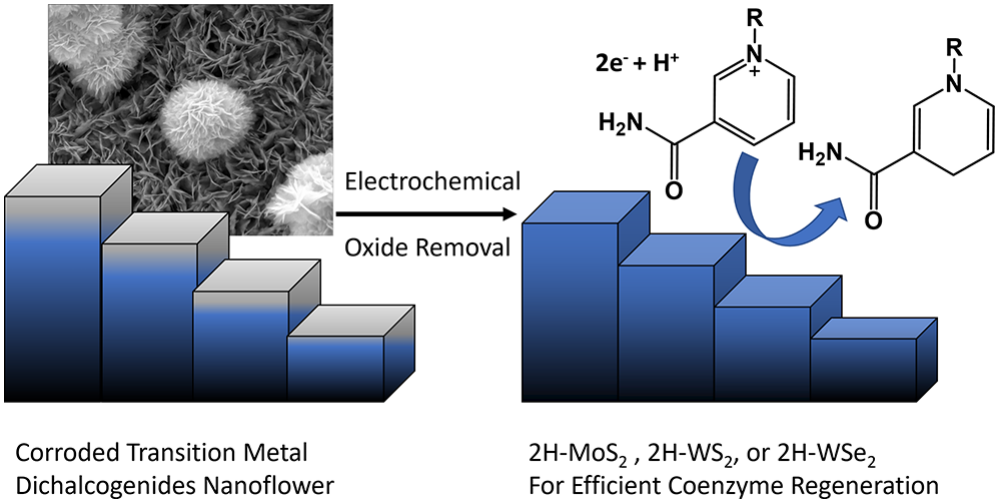

In the past 20 years, enzymatic conversions have been intensely examined as a practical and environmentally friendly alternative to traditional organocatalytic conversions for chemicals and pharmaceutical intermediate production. Out of all commercial enzymes, more than one-fourth are oxidoreductases that operate in tandem with coenzymes, typically nicotinamide adenine dinucleotide (NADH) or nicotinamide adenine dinucleotide phosphate (NADPH). Enzymes utilize coenzymes as a source for electrons, protons, or holes. Unfortunately, coenzymes can be exorbitant; thus, recycling coenzymes is paramount to establishing a sustainable and affordable cell-free enzymatic catalyst system. Herein, cost-effective transition metal dichalcogenides (TMDCs), 2H-MoS_2_, 2H-WS_2_, and 2H-WSe_2,_ were employed for the first time for direct electrochemical reduction of NAD^+^ to the active form of the NADH (1,4-NADH). Of the three TMDCs, 2H-WSe_2_ shows optimal activity, producing 1,4 NADH at a rate of 6.5 μmol cm^–2^ h^–1^ and a faradaic efficiency of 45% at −0.8 V vs Ag/AgCl. Interestingly, a self-induced surface reorganization process was identified, where the native surface oxide grown in the air was spontaneously removed in the electrochemical process, resulting in the activation of TMDCs.

## Introduction

In 2017, the chemical industry directly contributed $1.1 trillion and 15 million jobs to the global economy by producing essential chemicals such as NH_3_, methanol, and plastics, which are imperative for the modern world.^1^ Ninety percent of such chemical production is usually accomplished via catalytic conversions, employing heterogeneous or homogeneous catalysts.^1^ Nonetheless, catalytic processes in the industry tend to be energy-intensive, requiring substantial input of heat and pressure. A more cost-effective and sustainable approach must be developed, deviating from the current high energy-demanding process. In this regard, cell-free enzymatic systems are a contender with the capacity to create chemicals and pharmaceuticals under mild conditions.^2^ However, due to the high cost of coenzymes, like NADH, the capability to extend their eco-cycle via regeneration is critically important to meet the requirement for the potential scalability of enzyme catalysis.^3^ NADH is of prime interest due to its utilization in almost every living organism and its critical role in numerous cell-free enzymatic pathways.^2^ For instance, NADH has been used in the generation of both starch and methanol from CO_2_.^2,4,5^ Thus far, significant efforts have been put into regenerating coenzymes through complementary enzymatic pathways, homogeneous catalysts, heterogeneous catalysts, photocatalysts, electrochemical (EC) methods, and photoelectrochemical (PEC) methods.^6,7^ The use of an additional enzyme to facilitate the regeneration of NADH is found to be efficient and selective; however, extra enzymes can be costly and would have to be immobilized on a substrate to facilitate its separation from products.^8,9^ The use of homogeneous inorganic catalysts was found to be a promising method that can be easily separated from the products, with H_2_ as a reducing agent, Pt as a catalyst, and heat as an external energy resource.^3^ The photocatalytic regeneration of NADH is a greener method by exploiting light as a source of energy rather than electricity or heat. For instance, a promising photocatalytic system featuring a covalent organic framework (COF) with an immobilized electron mediator was shown to be stable and recyclable with an excellent NADH regeneration rate.^10^ However, photocatalytic conversions usually suffer from the issue of downstream separation, where the desired products can be hard to separate from byproducts, such as sacrificial reagents and photosensitizers. In addition, photocatalytic conversions often require the coupling of photosensitizers with rhodium(Rh)-based organometallic catalysts or enzymes to maintain a high selectivity for coenzyme regeneration.^9−13^ The pros and cons of each method have been extensively assessed in prior reviews.^6,14,15^ In this work, we will focus on sustainable electrochemical coenzyme regeneration, especially the rational design of catalysts to improve its electrochemical selectivity and efficiency.

The EC regeneration of NADH can be divided into indirect and direct regeneration (Scheme S1). The former utilizes an inorganic mediator, such as [Cp*Rh(III)(bpy)(H_2_O)]^2+^, which can selectively transfer a hydride to NAD^+^ for NADH regeneration.^16−19^ The reacted [Cp*Rh(III)(bpy)(H_2_O)]^2+^ can be catalytically recycled via direct EC regeneration or via electron transfer from an electron donor in solution, such as methyl viologen, which can be subsequently regenerated at a cathode.^20^ The main limitations of the indirect method are 2-fold. First, most homogeneous mediators are made of uneconomical precious metals, and second, homogeneous mediators commonly require downstream separation from the desired products and NADH, increasing production costs. While the downstream product separation can be addressed by immobilizing mediators via covalent functionalization or electrostatic interactions on conductive substrates, such as carbon nanotubes,^21,22^ mediators’ practical applications are still greatly hindered by their high cost.^16,17^ Direct EC regeneration also faces two consistent challenges, poor efficiency and low selectivity resulting from competing reactions. Coenzyme regeneration in the aqueous solution usually accompanies with the hydrogen evolution reaction (HER); the competing HER process will lower NADH regeneration’s faradaic efficiencies (FE). Herein, selectivity refers to the efficiency in generating the active 1,4-NADH isomer compared with other isomers and dimers, while FE describes the charge efficiency to generate the 1,4-NADH isomer. In direct EC NADH regeneration, if radical intermediate forms, a kinetically favorable dimerization or radical rearrangement could occur before the sluggish proton-coupled electron transfer.^23^ Thus, the reduction of NAD^+^ usually suffers from low selectivity for the desired 1,4-NADH. The issue of radical intermediates was surmounted with transition metal cathodes which would facilitate a concerted proton–electron transfer.^24^ However, such materials usually require large overpotentials, resulting in undesired HER, significantly reducing the efficiency of regenerating NADH. In summary, previous direct EC NADH regeneration overcame the issue of low selectivity but still struggles with poor efficiency.

In prior studies, the NAD^+^ reduction selectivity was dictated by the strength of the metal hydrogen (M-H) bond and the surface coverage of hydrogen on the metallic cathodes.^24−26^ Specifically, Ti, with the largest M-H strength, had the best selectivity (96%, −1 V vs Ag/AgCl). In contrast, Cd, with the weakest M-H strength, required a much larger energy input (−1.7 V vs Ag/AgCl) to achieve an optimal selectivity (93%). Interestingly, Co and Ni, which had medium M-H strengths, were both selective (82% and 92%, respectively) at a lower potential (−1.1 V vs Ag/AgCl).^25^ With the selective characteristic of Ni, Damian et al. further explored Ni nanoparticles (NPs) decorated on multiwalled carbon nanotubes (MCNT), which stabilized NAD^+^ intermediates through a van der Waals interaction.^27^ Ni NPs achieved selectivity of 98% through a proposed proton-coupled electron transfer followed by a subsequent electron transfer mechanism.^27^ Inspired by these works, we hypothesize that an ideal electrocatalyst for NADH regeneration should be able to stabilize the adsorbed H species and NAD^+^ intermediates through van der Waals interactions.

Transition metal dichalcogenides (TMDCs) meet the criteria for efficient NADH regeneration. Their edge sites can readily bond to hydrogen, with chalcogenide layers stacking together due to van der Waals interactions.^28^ The semiconductive 2H phases of molybdenum disulfide (MoS_2_), tungsten disulfide (WS_2_), and tungsten diselenide (WSe_2_) were chosen due to their ability to adsorb hydrogen, low toxicity, excellent biocompatibility, and economic viability.^29^ The structure and morphology of each electrocatalyst were characterized by Raman spectroscopy, X-ray photoelectron spectroscopy (XPS), and scanning electron microscopy (SEM). The rate, efficiency, and selectivity of NAD^+^ reduction to 1,4-NADH were determined by NMR, UV–vis, and electrochemical methods. It has been found that 2H-WSe_2_ selectively reduces NAD^+^ to 1,4-NADH at a rate of 6.5 μmol cm^–2^ h^–1^, with an FE of 45% at −0.8 V vs Ag/AgCl. This value is superior to the precious metal decorated PEC system (Pt decorated GaAs photoelectrode), in which NADH can be regenerated at −0.75 V vs Ag/AgCl with a FE of 17%.^7^ The selectivity, FE, and rates of current direct and indirect methods to reduce NAD^+^ to 1,4-NADH are compared and provided in Table S1. Further, it was found that the TMDC surface can be self-activated under the NAD^+^ reduction condition. In this process, a naturally grown surface oxide will inhibit the NAD^+^ reduction initially, and this inert oxide layer will be removed spontaneously in the electrochemical process.

## Material Characterization

**Figure 1:**
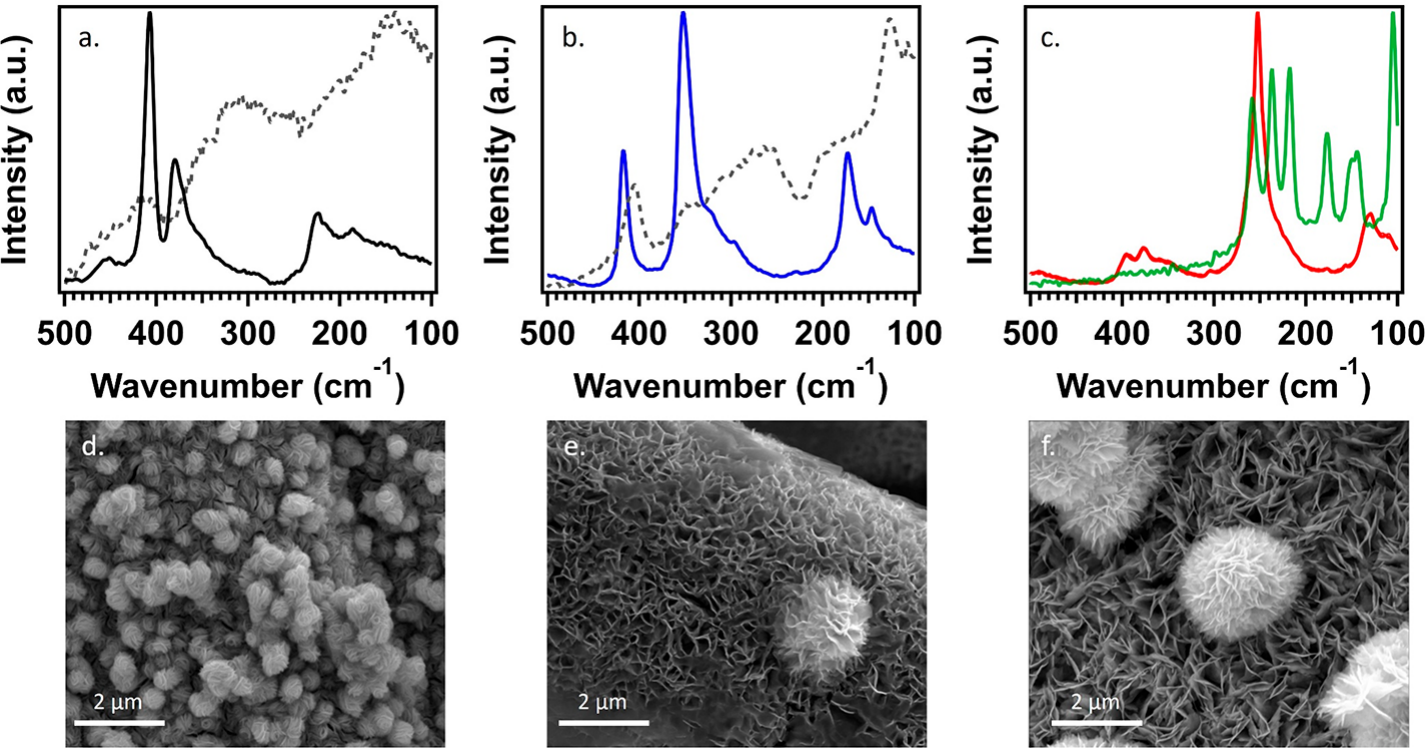
Raman spectra of (a) 2H-MoS_2_ (black) and the fabricated amorphous MoS_*x*_ (dashed gray), (b) 2H-WS_2_ (blue) and the fabricated amorphous WS_*x*_ (dashed gray), and (c) 1T-WSe_2_ (green) and 2H-WSe_2_ (red). SEM micrographs of (d) 2H-MoS_2_, (e) 2H-WS_2_, and (f) 2H-WSe_2_.

MoS_2_ was synthesized via a solvothermal microwave method directly onto carbon fiber paper (CFP), while WS_2_ and WSe_2_ were grown via conventional solvothermal syntheses (Schemes S2–S4). Each substrate underwent a second-step thermal treatment to yield the desired semiconductive 2H phase. Raman spectra of each TMDC were collected to confirm their chemical structures and phase before and after the thermal annealing process. Before a second-step thermal treatment, an amorphous MoS_*x*_, WS_*x*_, and metastable 1T-WSe_2_ were initially obtained (Figure 1a, b). The Raman spectra of 1T-WSe_2_ were consistent with previous literature (Figure 1 c).^30,31^ Upon thermal annealing, the out-of-plane A_1g_ vibration and in-plane E^1^_2g_ vibration can be seen for 2H-MoS_2_, 2H-WS_2_, and 2H-WSe_2_ (Figure 1a–c and Table S2), which confirm the successful formation of 2H-TMDCs.^32,33^ Herein, 2H-MoS_2_, 2H-WS_2_, and 2H-WSe_2_ all demonstrated uniform nanoflower structures as shown by the scanning electron microscopy (SEM) (Figure 1d, e). MoS_2_ generated from the solvothermal microwave method shows the formation of smaller nanoflowers, but the petals’ width was similar. The bare CFP without TMDC nanoflowers as a control is shown in Figure S1.

## Electrochemical Properties

2H-MoS_2_, 2H-WS_2_, and 2H-WSe_2_ were characterized electrochemically via linear sweep voltammograms and bulk electrolysis to assess the electrochemical performance and durability of catalysts. Linear sweep voltammograms were conducted under 0.1 M phosphate buffer solution (PBS) and 0.5 M H_2_SO_4_ (Figure S2) to determine the onset potential and overpotential of catalysts (Table S3). H_2_SO_4_ was employed in this work to investigate the active edge sites and compared TMDCs’ HER performance to prior literature, while PBS was used for NADH regeneration. Herein, the onset potential is the potential at which faradaic reactions begin (at 0.1 mA cm^–2^), and the overpotential is the additional potential beyond the thermodynamic requirement to drive the desired reaction, which for this purpose is the overpotential of HER. It was found that each TMDC showed decent HER activity in acidic media, where 2H-WS_2_ showed the best HER performance (Figure S2).^34−36^ As HER catalysts, TMDCs were less active than in the prior work.^36^ For instance, in this work, 2H-MoS_2_ had a 325 mV overpotential (10 mA cm^–2^) without *iR*-correction while previously reported stepped-edge 2H-MoS_2_ in 0.5 M H_2_SO_4_ produced an overpotential of 104 mV with *iR*-correction.^36^ The electrodes’ double-layer capacitance (*C*_dl_) was determined from the current response to the changed scan rate in a nonfaradaic region of cyclic voltammograms, where the ECSA of 2H-MoS_2_ (2.55 mF) was found to be significantly lower than those of 2H-WSe_2_ (7.40 mF) and 2H-WS_2_ (13.5 mF) (Table S3). To assess the chemical durability, electrolysis was conducted from −0.7 V to −1.2 V on 2H-MoS_2_ and 2H-WSe_2_ (Figures S3, S4, and S5). The TMDCs appeared to be stable in the deoxygenated PBS solution with a slight decrease in current density in the first 100 s of electrolysis, originating from the removal of trace surface oxides discussed in the section on TMDC activation.

## NAD^+^ Reduction

To assess NAD^+^ regeneration activity, electrochemical experiments were conducted in an H-cell with a constant N_2_ flow at the cathode to provide an oxygen-free environment. Three-electrode configuration was adopted where TMDCs on CFP, Ag/AgCl, and carbon plate were used as the working, reference, and counter electrodes, respectively. The direct electrochemical reduction of NAD^+^ to 1,4-NADH was optimized to minimize the competing HER and other side reactions, such as the formation of the inactive 1,6-NADH isomer, 4′4′-dimer, 4′6′-dimer, and 6′6′-dimer (Scheme S5). The TMDCs, activated before use, showed an increased selectivity consistent with a concerted two-electron one proton transfer pathway, as shown in Scheme S6.

**Figure 2:**
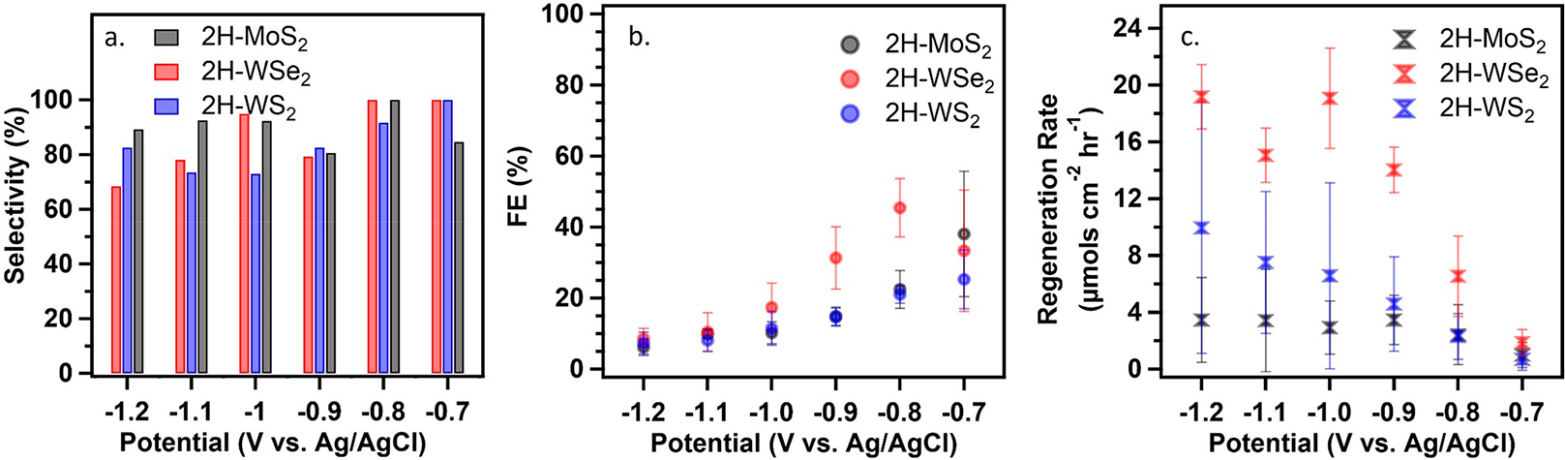
(a) Selectivity, (b) faradaic efficiency (FE), and (c) rate of NADH regeneration on 2H-MoS_2,_ 2H-WS_2_, and 2H-WSe_2_ at various potentials. All experiments were conducted after activation of TMDC in 1.0 mM NAD^+^ 0.1 M PBS at pH 7 with a carbon plate counter electrode and a Ag/AgCl reference electrode. Averages and error bars were taken from at least three separate measurements.

Initially, *in situ* grown 2H-MoS_2_ was a promising candidate with a FE of 22% at −0.8 V (Figure S6). It was initially hypothesized that this edge-rich MoS_2_ could convert NAD^+^ to NADH efficiently due to its favorable proton adsorption and adsorption of NAD^+^ through Van der Waal forces. However, this explanation does not elucidate why NADH could be produced at low potentials. Herein, we hypothesize that the hard–soft acid–base (HSAB) theory might explain the improved selectivity and reduced energy input for TMDCs.^37−39^ Wherein the adsorbed hydrogen atom on MoS_2_ is expected to function as a soft base, NAD^+^ would act as soft acid, reducing NAD^+^ to 1,4-NADH. In comparison, while transition metal catalysts used to date have been selective, they suffer from low efficiency due to the requirement of high overpotentials where HER is dominant.^24,27^ Hydrogen from transition metal are softer bases than that of the hydrogen from TMDCs while NAD^+^ is a soft acid; this mismatch would result in low transition metal conversion efficiencies (Table S1). In nature, NAD^+^ typically abstracts a proton from an organic substrate rather than from a transition metal.^40^ In an endeavor to assess this application of HSAB theory to direct electrochemical generation of NADH, a series of TMDCs was evaluated. The efficiency, selectivity, and rates of 1,4-NADH regeneration were determined for 2H-MoS_2_, 2H-WS_2_, and 2H-WSe_2_ (Figure 2).

The potential range used to assess the performance of the catalysts was selected based on the following selection criteria. As the potential becomes more positive, NAD^+^ could be efficiently reduced, but its regeneration rate will become comparable with the decomposition rate of NADH, making the quantification of products challenging (Figure 2c).^41^ In contrast, when the potential becomes more negative, HER becomes dominant, and the benefits of using these TMDCs over other transition metal catalysts are diminished (Figure 2b). When reducing NAD^+^, NMR was utilized to determine the selectivity of 1,4-NADH, relative to the inactive 1,6-NADH isomer. The NMR of commercially available NAD^+^ and 1,4 NADH are given in Figure S7. In addition, the characteristic peaks of 1,4-NADH and 1,6-NADH can be observed at 6.77 and 6.97 ppm, respectively (Figure S8). The NAD^+^ peak at 9.16 ppm was observed to decrease while the electrolysis proceeded (Figure S9).^3,7,42^ It was found consistently that the efficiency and selectivity of catalysts maximized at −0.8 V vs Ag/AgCl. As the potential increased from −0.8 V to −1.2 V, all TMDCs demonstrated decreased selectivity (Figure 2a) and FEs (Figure 2b). Herein, 2H-MoS_2_ and 2H-WS_2_ were found to have similar selectivity, which can be attributed to the similar softness of S when hydrogen adsorbed on their surfaces. While both 2H-MoS_2_ and 2H-WS_2_ achieved near 100% selectivity at −0.7 V and −0.8 V, the rate of NADH regeneration on 2H-WS_2_ is higher at all potentials (Figure 2c). This phenomenon is attributed to its larger ECSA (Table S3). The 2H-WSe_2_ performance is expected to be different from the sulfides due to the softer nature of Se–H bonds. This is observed by the significantly larger FE of 2H-WSe_2_ compared to the sulfides and a substantial increase in the NADH regeneration rate of 2H-WSe_2_ compared with that of 2H-WS_2,_ which has a larger ECSA. The increased activity of 2H-WSe_2_ is attributed to the smaller bond strength of Se–H compared with S–H, indicating that Se provides more compatible hydrogen for NAD^+^.

**Figure 3:**
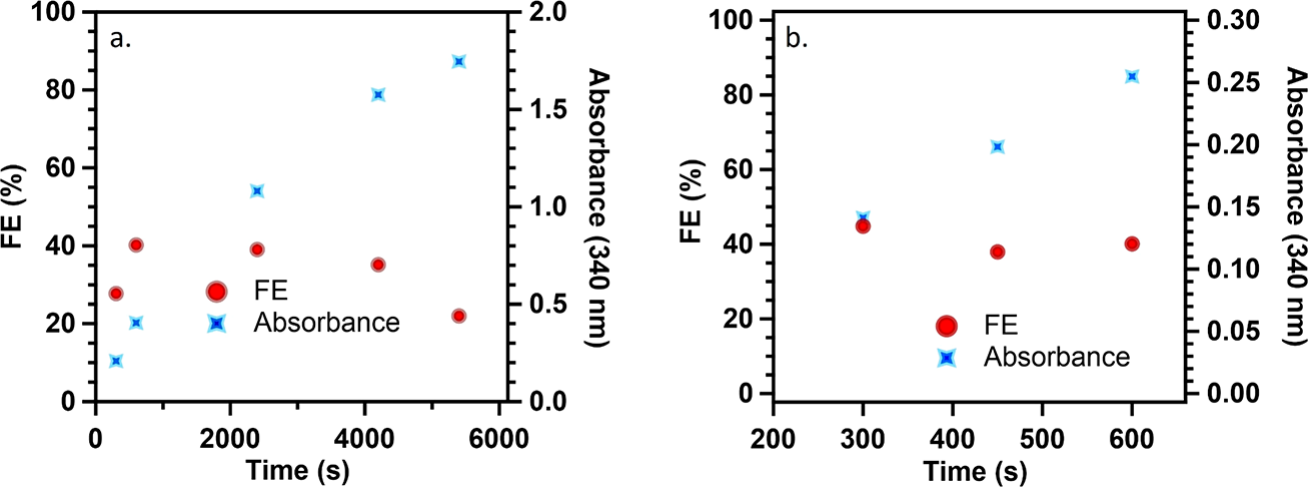
Relationship between faradaic efficiency and concentration of NAD^+^, shown (a) without electrochemical activation over 6000 s electrolysis and (b) after electrochemical activation. NAD^+^ was reduced at −0.8 V vs Ag/AgCl using a 2H-WSe_2_ working electrode; 3.00 mL of 1.0 mM NAD^+^ was used in the working electrode compartment of an H-cell, 0.1 M PBS supporting electrolyte at pH 6.91, carbon CE, Ag/AgCl RE, 100 scc min^–1^ N_2_ in the WE compartment.

When NAD^+^ regeneration proceeded, a decrease in current was observed over time. This phenomenon is accredited to the decreased concentration of NAD^+^. As the reaction proceeds, the concentration of NAD^+^ in the bulk solution and at the electrode interface decreases, resulting in a decrease FE toward coenzyme regeneration (Figure 3).

## TMDC Activation: Electrochemical Oxide Removal

While monitoring FE as a function of time, the FE was expected to decrease with time as NAD^+^ was reduced. Unexpectedly, TMDCs consistently experienced low FE initially, which would increase in the first few minutes while NAD^+^ reduction proceeded (Figure 3a). This initial improvement in FE was identified as a self-activation process, during which electrons are utilized to remove the surface oxide layer, which results in exposure of active sites of TMDCs (Scheme S7). During the electrochemical activation, hydrogen adsorbed to the surface oxides can act as harder bases, resulting in lower FEs. After the initial of catalysts’ activation stage, the FE becomes stable, followed by decreasing over time (Figure 3b).

**Figure 4:**
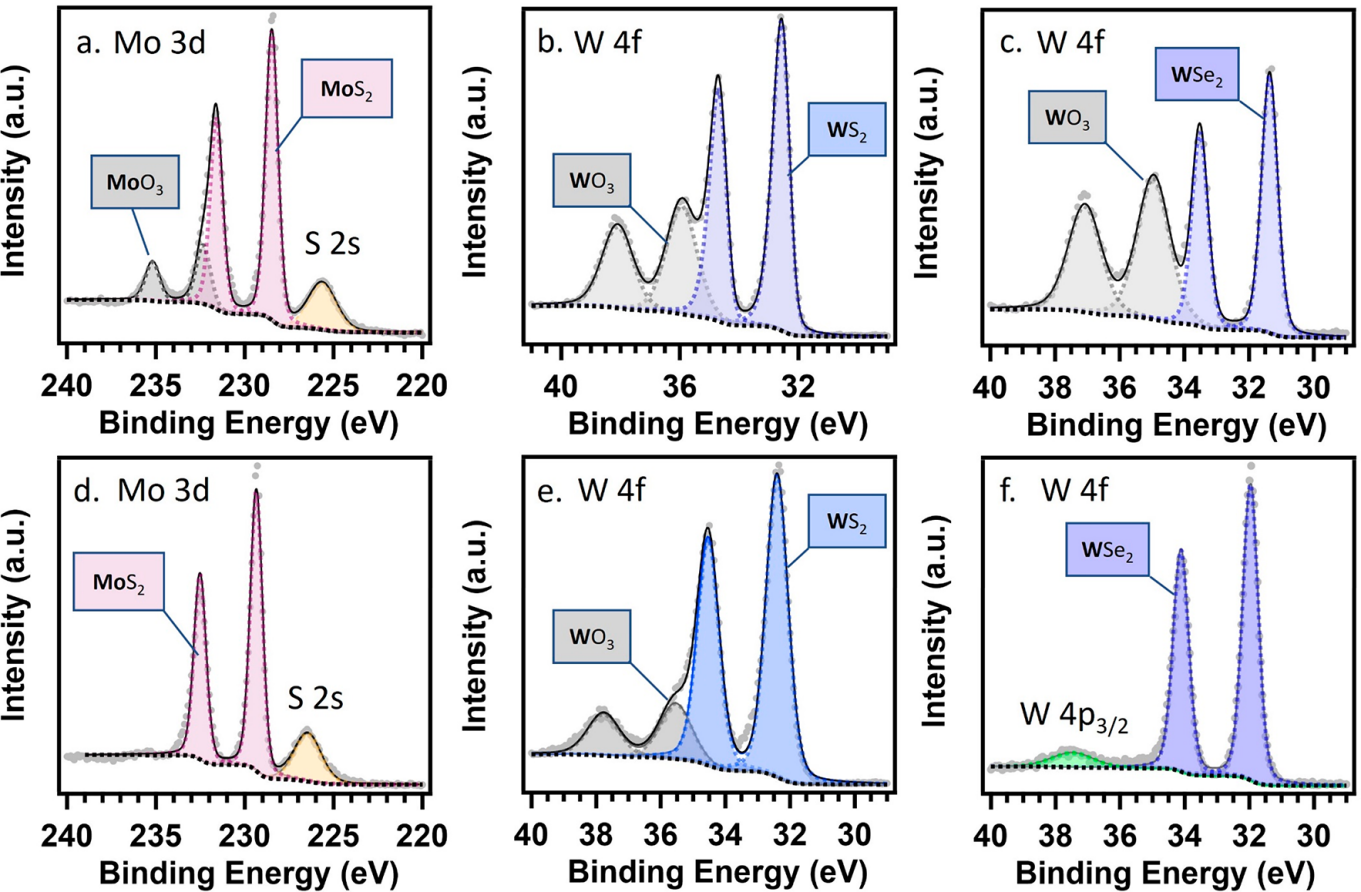
Detailed XPS spectra of metal core electrons collected (a–c) before and (d–f) after electrolysis for (a, d) 2H-MoS_2_, (b, e) 2H-WS_2_, and (c, f) 2H-WSe_2_. Electrolysis was conducted at −0.8 V vs Ag/AgCl in an H-cell with 0.1 M PBS electrolyte with N_2_ sparging for 1200 s.

To confirm the surface structure reorganization from electrolysis in these near-neutral conditions, X-ray photoelectron spectroscopy (XPS) was conducted after thermal annealing and after electrolysis. The survey spectra show the presence of transition metals, chalcogenides, carbon, and oxygen on the surface of the TMDCs after annealing (Figures S10, S11, and S12). In addition, after annealing, a mix of expected TMDCs and a native oxide layer with the assignment and peak positions (detailed in Table S4) was identified. For instance, from the Mo 3d spectra, the as-synthesized amorphous MoS_*x*_ consisted of a mixture of MoO_3_, MoS_*x*_, SO_4_^2–^, and S^0^ species (Figure S13), where the elemental S^0^ might be originated from the solvothermal synthesis. The presence of SO_4_^2–^ was identified by hexavalent S 2p doublet at 168 eV. Annealing the amorphous MoS_*x*_ yields 2H-MoS_2_ with a reduced quantity of MoO_3_ and SO_4_^2–^ (Figure 4a, Figure S14a). 2H-MoS_2_ was present as evidenced by the near stoichiometric ratio confirmed by Mo^4+^, with Mo 3d_5/2_ and 3d_5/2_ binding energies of 228.5 and 231.6 eV and S^2–^ S 2s at 225.6 eV, and S 2p peaks at 161.3 and 162.4 eV. Likewise, after annealing, 2H-WS_2_ was identified by the existence of W 4f_7/2_ and W 4f_5/2_ (Figure 4b) at 32.14 and 34.28 eV and S 2p_3/2_ and S 2p_1/2_ (Figure S14b) at 161.78 and 162.97 eV, respectively (Table S4).^43^ While not observed through Raman spectroscopy (Figure 1), trace amounts of MoO_3_ could be identified with XPS by the presence of Mo^6+^ 3d_3/2_ and Mo–O 1s (530.5 eV). Likewise, WO_3_ was identified to be grown on 2H-WS_2_ and 2H-WSe_2_ by the presence of W^6+^ 4f_7/2_ and 4f_5/2_ and W–O O 1s peak at ∼530.5 eV. The ratios of metal to dichalcogenide (Table S6) were quantified after annealing, where a near stoichiometric 1:2. The conversion of the conductive 1T-WSe_2_ to 2H-WSe_2_ was observed (Figures 4c, S14c, and S15), by the increase in binding energies of W^4+^ W 4f and Se^2–^ Se 3d peaks consistent with a shift from metallic to semiconductive phase (Table S4). Utilizing the native oxide layer, the binding energy separation of W^4+^ W 4f_7/2_ and W^6+^ W 4f_7/2_ further confirmed this phase transition, where binding energy separations were found to be 3.28 and 4.24 eV for the 2H-WSe_2_ and 1T-WSe_2_, respectively, consistent with the prior literature.^30,44,45^

For 2H-MoS_2_, 2H-WS_2_, and 2H-WSe_2,_ the ratio of Mo^4+^ to Mo^6+^ or W^4+^ to W^6+^ increased dramatically after electrolysis (Table S6), indicating that a structural change occurred at the surface during electrolysis. The presence of oxides (MoO_3_ on MoS_2_, and WO_3_ on WS_2_ and WSe_2_) was identified before electrolysis, by the Mo^6+^ Mo 3d_3/2_ and the W^6+^ W 4f_7/2_ and at ∼236 eV and ∼36 eV, respectively (Figure 4). The Mo^6+^ species was absent from the 2H-MoS_2_ surface after electrolysis (Figure 4d). The decrease in the presence of lattice oxygen after electrochemical activation is also apparent from the decreased O 1s peak at ∼530.5 eV (Figure S16). Similarly, 2H-WSe_2_ showed a significant decrease in surface oxides after electrolysis as compared to before electrolysis (Figure 4f). After electrolysis, the structure of 2H-MoS_2_, 2H-WS_2_, and 2H-WSe_2_ were retained at the surface, as evidenced by the similar binding energies observed before and after electrolysis (Table S7 and Table S8). 2H-WS_2_ showed a substantial decrease in surface oxides after electrolysis (Figure 4e). After the electrochemical etching of the native oxide layer, the catalysts are activated, shown by a constant FE during electrochemical NAD^+^ reduction (Figure 3b).

From *ex situ* XPS results, the electrochemical removal of surface oxides can be correlated to the enhanced FE observed as the NAD^+^ reduction proceeding. *Ex situ* Raman spectroscopy was employed to provide further evidence that the oxides could be removed through electrochemical methods. When fabricated, no MoO_3_ or WO_3_ could be detected via Raman spectroscopy. However, it was found that after eight months of storage in the air, surface oxidation of 2H-MoS_2_ was confirmed by the O–Mo–O wagging mode at 281 cm^–1^, a symmetric stretch at 820 cm^–1^ and an asymmetric stretch at 992 cm^–1^ (Figure S17).^46,47^ Further, the LA(M) phonon mode associated with the presence defects at the edges of 2H-MoS_2_ decreased after exposure to air for eight months, indicating that oxidation is occurring at the edges of MoS_2_ sheets, blocking the activation sites for NADH regeneration.^48^ This thick oxide layer can be removed through cathodic linear sweep voltammograms or electrolysis in neutral PBS without NAD^+^, similar to the electrolysis result with NAD^+^, yielding a surface nearly identical to its nonoxidized state (Figure S18). The electrochemical oxide removal in a neutral phosphate buffer solution with and without the coenzyme could activate the TMDC electrodes for NAD^+^ reduction. Once activated, the TMDCs were found to be stable after four cycles of NAD^+^ reduction experiments (Figure S19a); additionally, corroded electrodes could be reactivated up to five times with little change of FEs (Figure S19b).

## Conclusion

To broaden the direct electrochemical coenzyme regeneration field, a series of TMDCs were exploited as electrocatalysts to convert NAD^+^ to 1,4-NADH. 2H-WSe_2_ had the optimal performance for the regeneration of NADH at a modest potential −0.8 V vs Ag/AgCl, outperforming 2H-MoS_2_ and 2H-WS_2_ in terms of NADH regeneration rate and FE. These materials excelled in the performance of regenerating NADH because of their stability and inherit activity which is attributed to the soft acid–base interaction between NAD^+^ and the active chalcogenide sites of the TMDCs. Further, it was found that the TMDC performance was initially limited due to the presence of trace surface oxides which significantly impede coenzyme regeneration; after electrochemical etching of the oxide, the activated surfaces can efficiently regenerate coenzymes.

## Experimental Section

### Materials

Potassium phosphate monobasic ≥99.0%, dipotassium phosphate 98.0 to 102.0%, acetone 99.5%, methanol 99.9%, and sulfuric acid ≥97.0% were purchased from Fisher Scientific and used without further purification. Toray carbon paper TGP-H-60 (CFP) was purchased from Advanced Instruments. Ethanol, 200 proof (100%), was purchased from Decon Laboratories and used without further purification. Sodium molybdenum oxide anhydrous ≥99.2% and sodium tungsten oxide dihydrate 99.0 to 101.0% were purchased from Alfa Aesar and used without further purification. Selenium powder ≥99.5%, Urea ≥98%, and sulfur powder ≥99% were purchased from Sigma-Aldrich and used without further purification. Thioacetamide 99%+ was purchased from Acros Organics and used without further purification.

### Catalyst Fabrication

Carbon fiber paper (CFP) was cleaned via washing and sonicating in an acetone solution followed by ethanol, water, and dried at 80 °C before use. For 2H-MoS_2_ fabrication, the cleaned CFP substrate was added to a 10 mL monowave G10 vial, filled with 4.0 mL DI water, and sparged with N_2_ for 10 min. The vial was firmly tapped against the benchtop to dislodge bubbles present on the hydrophobic CFP. Anhydrous sodium molybdate (∼82 mg) was added and sparged with N_2_ for 10 min, thioacetamide (∼22 mg) was added and sparged for 10 min, after which the vial was sealed with a PTFE septum. The microwave-solvothermal growth of MoS_2_ was conducted with an Anton Paar Monowave 400 and included a four-step process. First, the solution was heated to 175 °C for 5 min, then heated to 200 °C for 15 min, followed by holding at 200 °C for 15 min. Lastly, the sample was cooled naturally to 50 °C. Heating the solution over time, rather than using the instruments as-fast-as-possible method, was essential to avoid exceeding the instruments’ maximum allowed pressure and sparking on the conductive CFP. Amorphous MoS_*x*_ was converted to 2H-MoS_2_ by heating the sample in the DI water at 215 °C for 15 min with microwave radiation, after which the water had a strong thiol odor.

2H-WSe_2_ with nanoflower morphology was fabricated with a method adapted from previous literature.^49,50^ Elemental selenium (0.2748 g) and Na_2_WO_4_ (0.5122 g) were dissolved in 26.0 mL of dimethylformamide (DMF) and allowed to stir for 15 min, with N_2_ sparging, after which 0.0510 g of NaBH_4_ was slowly added and stirred for 15 min. Next, 16.0 mL of water was added, producing a deep red solution. Ten milliliters of solution was added to an autoclave containing a piece of CFP (1 cm × 2 cm), sparged with N_2_ for 10 min, and then sealed. The sealed autoclave was heated to 200 °C for 48 h and cooled naturally to afford 1T-WSe_2_. This method was employed to grow WS_2_, using elemental sulfur rather than selenium as a precursor, and produced an amorphous WS_*x*_ product. Both 1T-WSe_2_ and WS_*x*_ were annealed at 400 °C for 30 min under nitrogen flow to afford 2H-WSe_2_ and 2H-WS_2_, respectively. The TMDC-decorated CFP was rinsed with DMF, followed by 5 min of sonication in H_2_O three times to remove any physioadsorbed materials and unreacted salts.

### Additional Notes on Sample Preparation and Intermediates

Bulk powders obtained from the synthesis of each TMDC were found to be in the 2H phase without further annealing (Figure S20). When WS_2_ or WSe_2_ were synthesized without N_2_ sparging, the bulk powder produced in the hydrothermal reactor consisted primarily of WO_3_ (Figure S21). WO_3_ was identified by O–W–O stretching observed at 806 cm^–1^, W–O stretching at 686 cm^–1^, and O–W–O bending at 260 cm^–1^.^51,52^ Using this method to attempt to fabricate 2H-WS_2_ A_1g_ and E^1^_2g_ peaks could also be identified but were minor in size. However, for the case of WSe_2_ neither 1T-WSe_2_ or 2H-WSe_2_ peaks were detected.

### Electrochemical Methods

Electrochemical experiments were conducted using an Autolab potentiostat. An H-cell with mesoporous glass frits between each electrode was used to minimize dissolved oxygen concentrations in the working electrode compartment during bulk electrolysis and to prevent NADH oxidation on the anode. The working electrode compartment was sparged with N_2_ for a minimum of 10 min at a rate of 100 scc min^–1^ before all electrochemical tests unless noted. Voltammograms were collected in a quiescent solution, while bulk electrolysis was conducted with N_2_ sparging. Sparging during NAD^+^ reduction reduced the diffusion limitations and protected the TMDCs from oxidation by dissolved oxygen.^53^

The electrochemical activation of the TMDCs, which removed surface oxides, was conducted in an H-cell with 0.1 M PBS pH 7 supporting electrolyte. The electrochemical activation of TMDCs was carried out before NAD^+^ reduction. It was found that during the activation process the solution in the working electrode compartment had a thiol odor, presumed to be H_2_S or SO_2_. The activation should be carried out under a ventilation snorkel or fume hood.

While TMDC electrodes were found to be reusable for several experiments with reactivation, there were two common modes of mechanical failure. First, the CFP support was prone to accidental snapping if it was not handled with care. Second, the end of the TMDC modified electrode, if accidently connected to working electrode holder, would experience physical abrasion with use.

UV–vis and NMR spectroscopy were used to determine the efficiency and selectivity. From UV–vis spectra, the peak absorbance at 340 nm was utilized to determine the yield of 1,4-NADH and its isomer and dimers. It is of note that, in addition to the competition from HER, the regeneration of NADH is usually complicated by the instability of NADH itself. NADH is prone to degrade depending on the ions in solution, pH, and temperature.^54−56^ NADH is chemically unstable and is known to react with phosphate ions slowly.^41,54^ Electrolysis was conducted from −0.7 V to −1.2 V until the absorbance at 340 nm was ≥1. When absorbance was ≤1, it was challenging to obtain reliable NMR spectra for comparing the ratio of 1,4-NADH to 1,6-NADH isomers. It is of note that at lower potentials, −0.6 V vs Ag/AgCl, NADH could be detected with UV–vis spectroscopy, but due to the inherent instability of the reduced coenzyme and slow rate of generation, an NMR suitable concentration was never achieved. Without the determination of selectivity, the rate of 1,4-NADH generation and FE at −0.6 V vs Ag/AgCl could not be credibly quantified. Out of concern that 1,6-NADH was not observed at lower quantities due to the lack of reduced NAD^+^, selectivity measurements were only obtained after at least one-third quantity of NAD^+^ was reduced.

All potentials were converted to RHE using eq 1, where *E*_Ag/AgCl_ is the experimental electrode potential used vs Ag/AgCl and *E*°_Ag/AgCl_ is +0.209 V (the standard electrode potential of an Ag/AgCl electrode with 3 M KCl electrolyte). The overpotential is defined as the additional potential (vs RHE) beyond the thermodynamic requirement, commonly referenced to the current density of 10 mA cm^–2^.
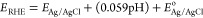
1

### UV–Vis Spectroscopy

All UV–vis experiments were conducted using an Agilent Carry 60. The appropriate concentration of NAD^+^ in 0.1 M PBS was used as a baseline for all experiments. Samples in a quartz cuvette were tested immediately at the end of electrolysis and scanned at a rate of 600 nm s^–1^ from 600 to 280 nm with a data collection interval of 0.1 nm.

### NMR

NMR samples were prepared with 90% electrolyte from working electrode compartment of H-cell and 10% D_2_O solutions. Data was collected on a Varian 400 using a water suppression pulse sequence. All NADH NMR data were collected immediately after the cessation of their respective experiments to minimize the decomposition of products.

### Determining Selectivity, Rate of 1,4-NADH Generation, and FE

Selectivity was determined from NMR, using the ratio of 1,4-NADH and 1,6-NADH. The FE was determined from NMR, UV–vis, and electrochemical results, shown in eq 2. The TMDCs were activated before experiments, in which reported FEs, rates, and selectivity were determined. The total concentration of 1,4 and 1,6-NADH would be identified by using Beer–Lambert Law with the volume of solution known, the product yield was determined by multiplying moles of NADH by the selectivity of 1,4-NADH, which is then divided by the theoretical moles of NADH that could be calculated from the charge passed during the electrolysis process. The rate of NADH regeneration and the selectivity were determined by monitoring the absorbance at 340 nm via UV–vis spectroscopy.
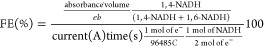
2

### Scanning Electron Microscopy (SEM)

Samples adhered to SEM stubs with carbon tape. Micrographs were collected using an FEI Quanta 450 FEG scanning electron microscope. The width of the TMDC nanoflower petals was determined with ImageJ software using high-quality, high-magnification images (Figure S22).

### Raman Spectroscopy

Raman spectra were collected using a ThermoScientific DXR Raman microscope. The surface of each sample was focused on with a 10× optical lens, and measurements were carried out with a 532 nm argon laser, 50 μm pinhole, and 900 lines/mm grating. Spectra were collected using 5 s exposure time with 12 background exposures followed by 12 sample exposures.

Regarding *ex situ* Raman spectroscopy, it should be noted that the samples available for testing were limited to partially oxidized surfaces. It was found that *in situ* grown TMDCs oxidized slowly, requiring several months to grow enough to detect with Raman spectroscopy.

### X-ray Photoelectron Spectroscopy

All XPS spectra were collected on a PHI 5600 XPS system equipped with Monochromatic Al Kα 1486.6 eV X-ray source and Omni Focus III lens, with a 90° angle between the X-ray source and analyzer. Before data collection, the instrument was calibrated to Au 4f7/2, 84.00 eV, and Cu 2p3/2, 932.67 eV. The maximum base pressure was 5 × 10^–9^ Torr. Survey spectra were collected with a pass energy of 117.4 eV, a 1.0 eV step size, and a 50 μs dwell time. Detailed spectra were collected with a pass energy of 11.75 eV, a 0.05 eV step size, and a 150 μs dwell time.

XPS fittings were conducted in an Igor package. The following constraints were used: doublets had the same formula width half max (fwhm); p, d, and f doublets had relative areas of 1:2, 2:3, and 3:4, respectively. All detailed spectra had a Shirley background for their baseline. In fitting the metallic 1T-WSe_2_ data, asymmetry was allowed in its peaks shape for the W^4+^ W 4f doublet due to its metallic character.

Atomic ratios were determined with eq 3, where *n*_1_/*n*_2_ is the ratio between two chemical species, *I* is the integrated fitted area of the chemical species, and ASF is the atomic sensitivity factor of the core electron of the respective elements. Appropriate PHI Omni Focus III at 90° ASF area values were used. To determine the most accurate atomic ratios, the area was found from the peak fittings of each chemical species in the detailed spectra. Survey spectra were not used for quantification as the metal’s region would contain +6 oxides as well the +4 states, producing a systematic error of higher M:X ratios.
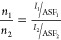
3

## Supporting Information Available

Supporting Information contains supplementary XPS spectra, SEM micrographs, Raman spectra, electrolysis data, NMR spectra, reaction schemes, XPS peak assignments, and Raman peak assignments (PDF)
