# Supplementary material for: Surface Reorganization of Transition Metal Dichalcogenide Nanoflowers for Efficient Electrochemical Coenzyme Regeneration
Source: ACS Appl Mater Interfaces. 2023 Jan 11;15(3):3925–33. doi: 10.1021/acsami.2c17483 (PMC9880950; doi:10.1021/acsami.2c17483)
Supplement: Supplementary file 1 — am2c17483_si_001.pdf [file am2c17483_si_001.pdf]

Supporting Information

Surface Reorganization of Transition Metal Dichalcogenides Nanoflowers for Efficient Electrochemical Coenzyme Regeneration

Nicholas Williams[1]; Karley Hahn[1]; Ryan Goodman[1]; Xiaowen Chen[2]; Jing Gu[1]*

1 Department of Chemistry and Biochemistry, San Diego State University, 5500 Campanile Drive, San Diego, CA, 92182, United States

2 Catalytic Carbon Transformation and Scale Up Center, National Renewable Energy Laboratory, 15013 Denver West Parkway, Golden, CO, 80401, United States

* Email: jgu@sdsu.edu

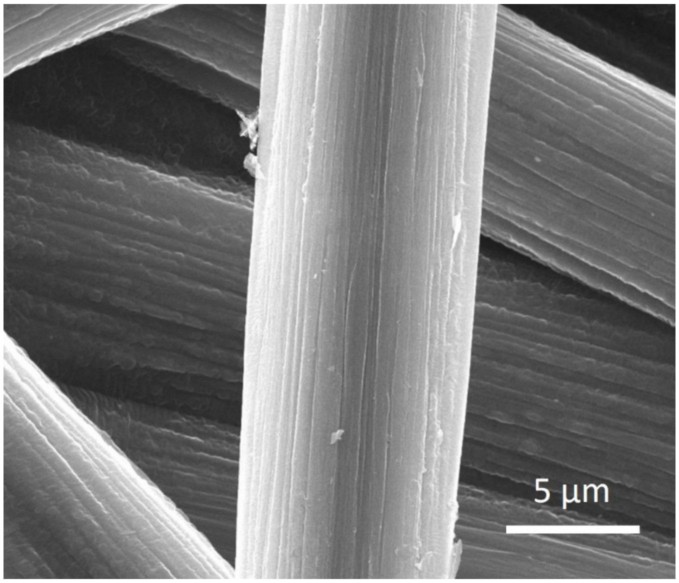

Figure S1: Micrograph of bare CFP prior to *in-situ* growth of TMDCs.

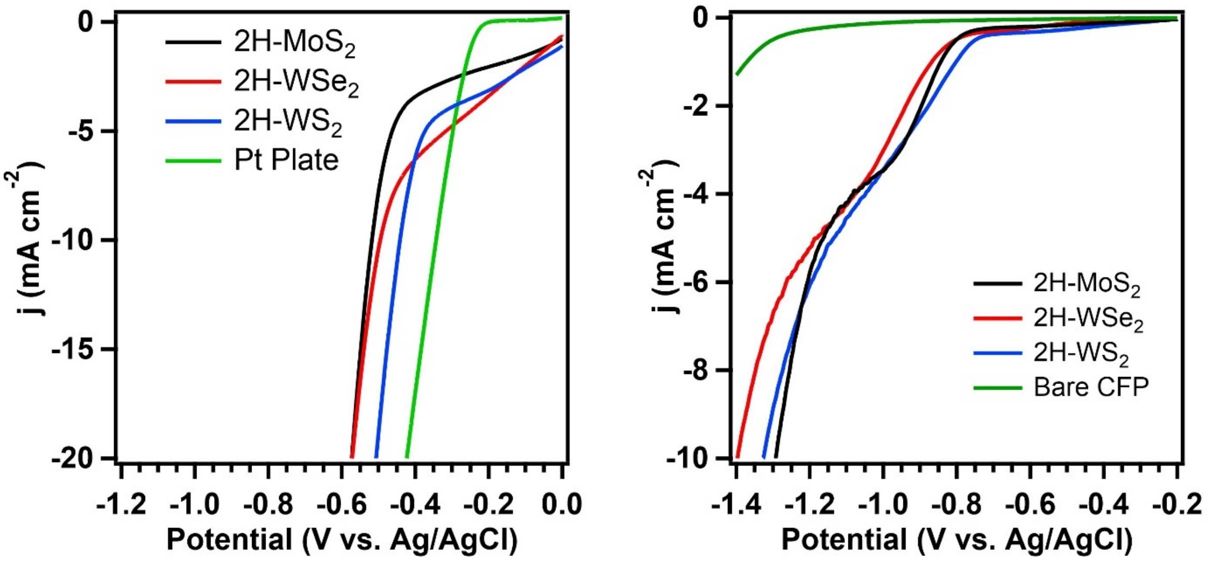

Figure S2: Linear sweep voltammograms (5 mV s^{-1}) of transition metal dichalcogenides in a 0.5 M H_2SO_4 (left) and a 0.1 M phosphate buffer solution (PBS, pH=6.91, right). N_2 was sparged through the electrolyte for 15 minutes before the data was collected.

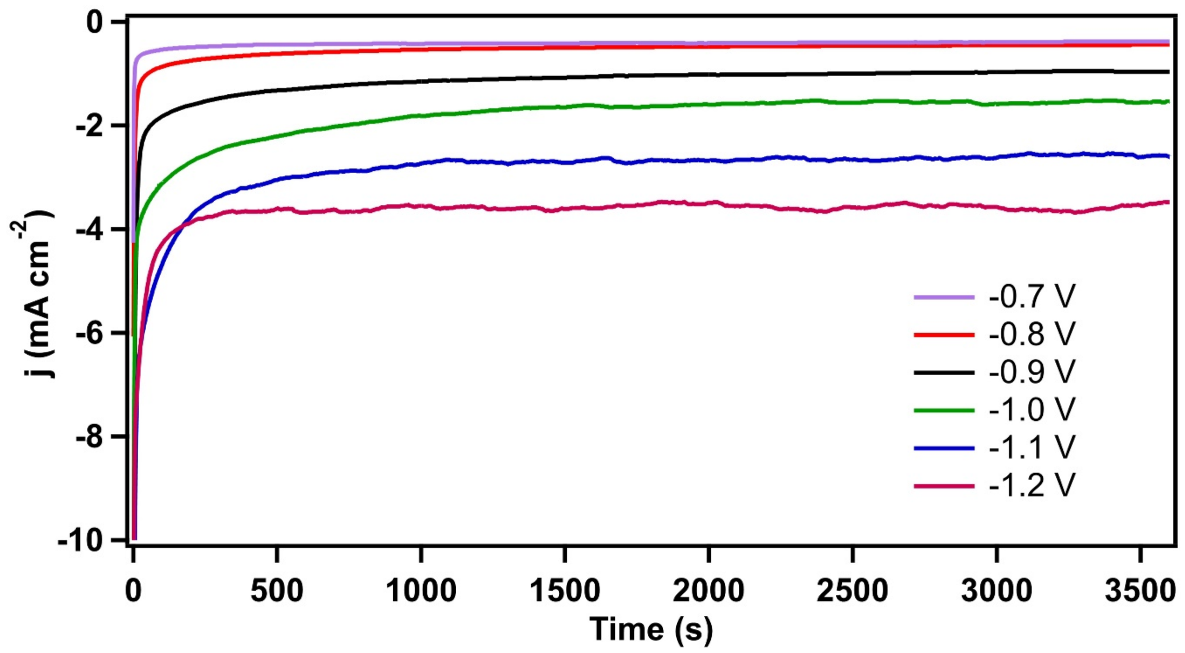

Figure S3: HER electrolysis at various potentials (vs. Ag/AgCl) using 2H-MoS_2 in 0.1 M PBS (pH=6.91) without the addition of coenzyme. Herein, carbon plate was used as the counter electrode; each test was performed with N_2 at a flow rate of 50 scc min^{-1} over the solution.

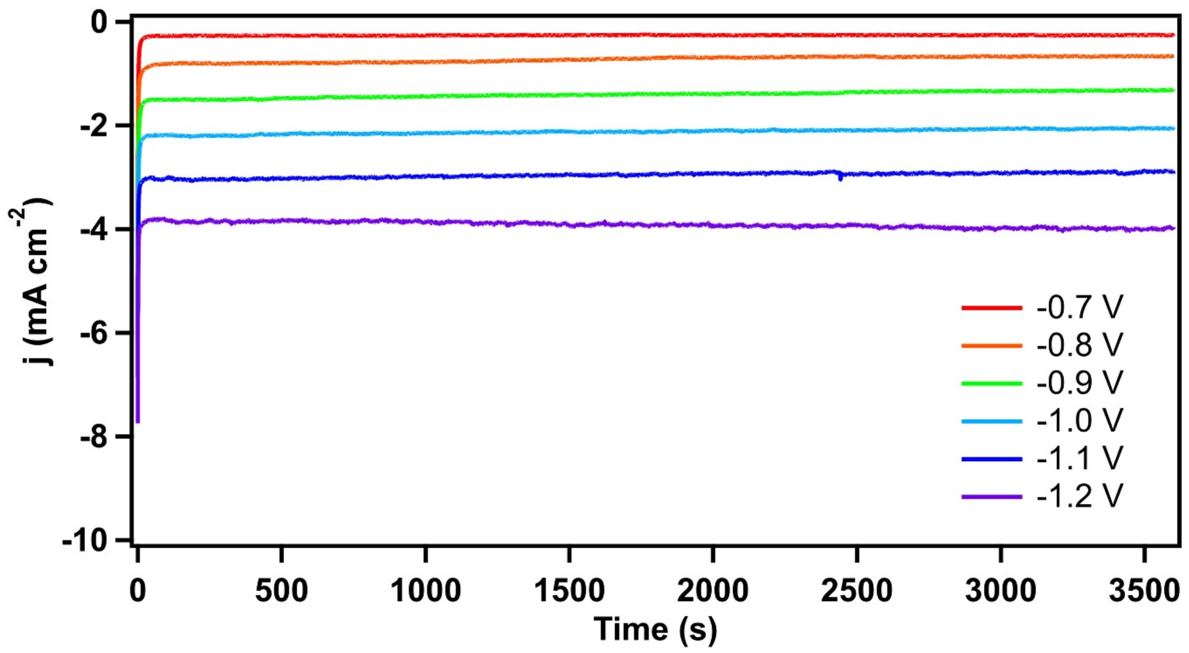

Figure S4: HER electrolysis at various potentials (vs. Ag/AgCl) using 2H-WS_2 in 0.1 M PBS (pH=6.91) without the addition of coenzyme. Herein, carbon plate was used as the counter electrode; each test was performed with N_2 at a flow rate of 50 scc min^{-1} over the solution.

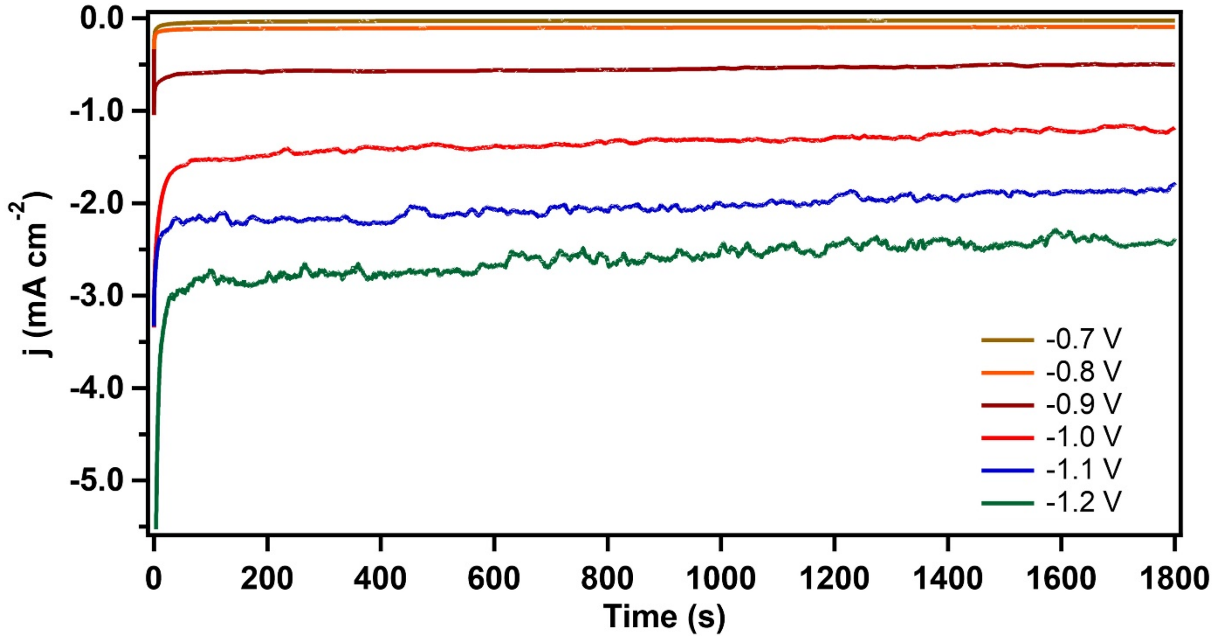

Figure S5: HER electrolysis at various potentials (vs. Ag/AgCl) using 2H-WSe_2 in 0.1 M PBS (pH=6.91) without the addition of coenzyme. Herein, carbon plate was used as the counter electrode; each test was performed with N_2 at a flow rate of 50 scc min^{-1} over the solution.

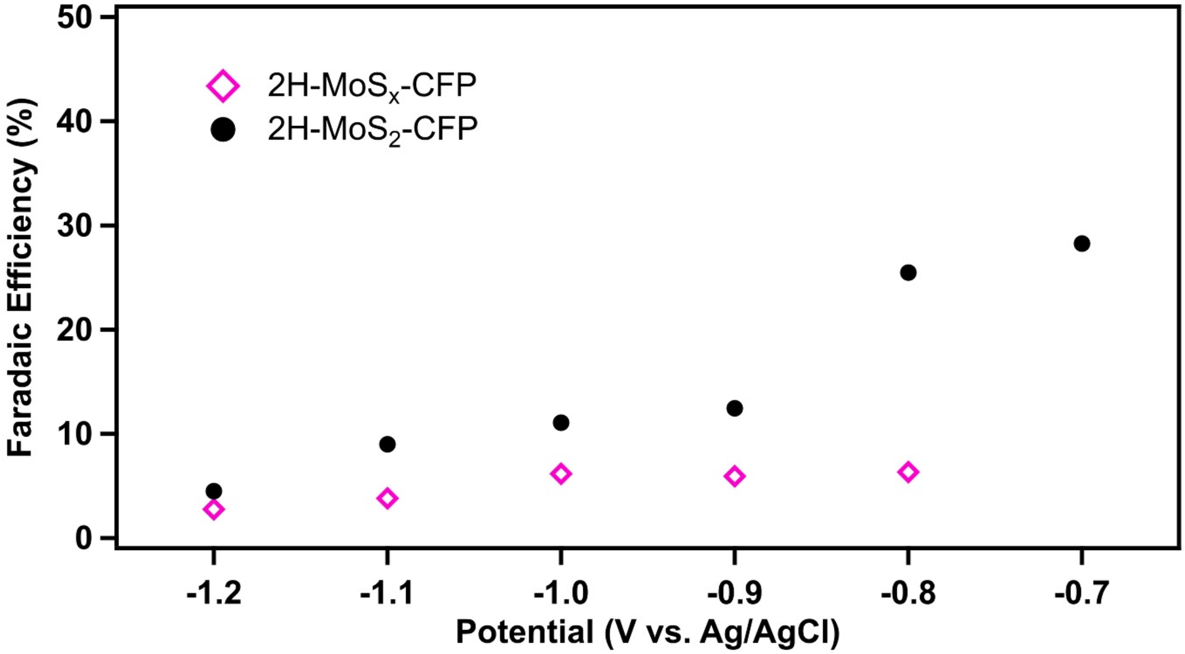

Figure S6: Faradaic efficiencies of 1,4 NADH regeneration at various potentials before and after thermal treatment of MoS_x to 2H-MoS_2. In this work, for amorphous MoS_x, FE was not reported at -0.7 V due to lack of the detectable product.

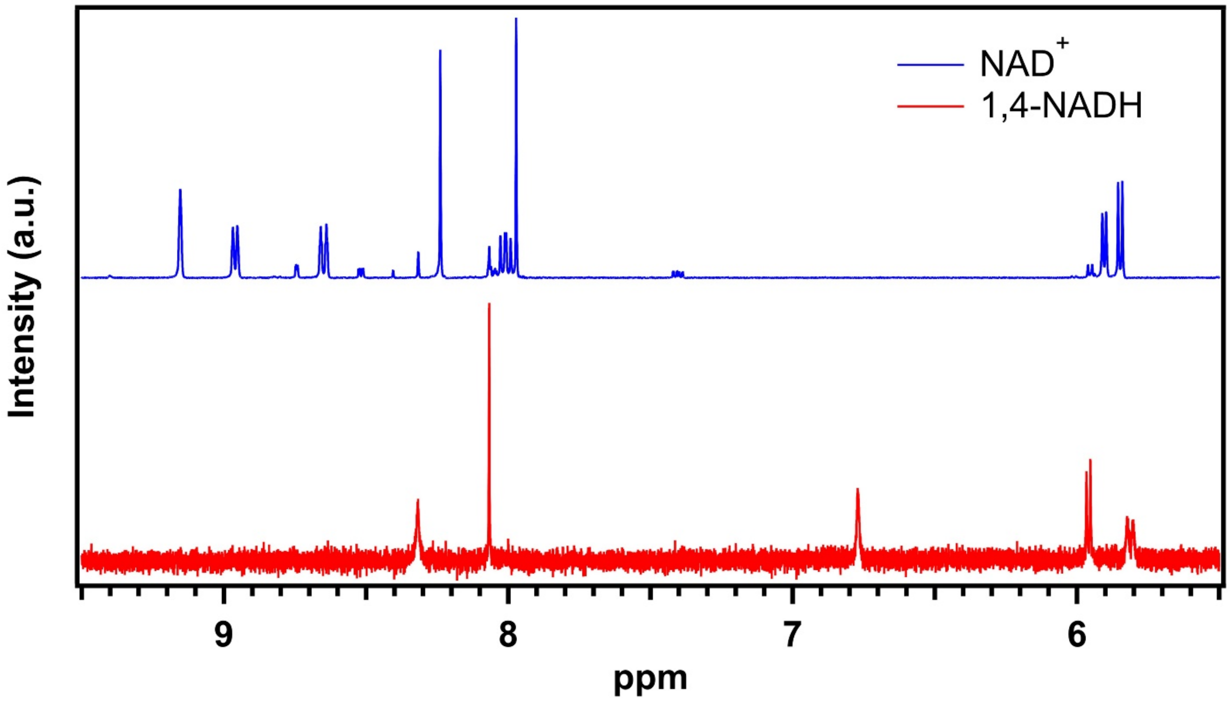

Figure S7: NMR spectra of commercial beta-Nicotinamide adenine dinucleotide in its oxidized and reduced forms (NAD^+ and 1,4 NADH, respectively). Spectra were collected with a ^1H pre-saturation method using a 90 to 10 ratios of 0.1 M PBS to D_2O.

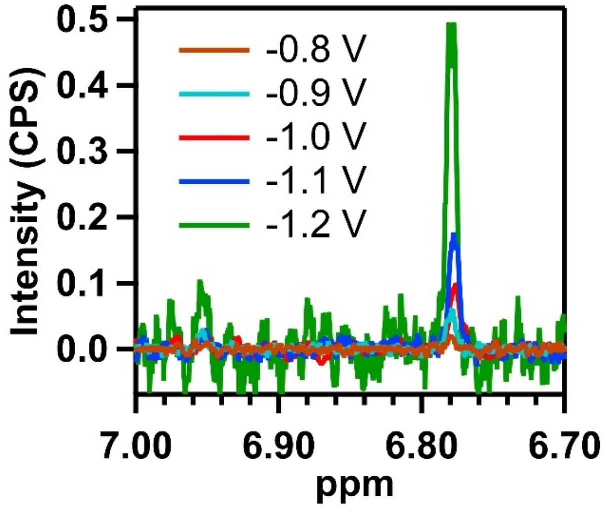

Figure S8: NMR spectra of 1,4 NADH with a characteristic peak at 6.77 ppm and a byproduct of 1,6 NADH with a characteristic peak at 6.95 ppm. Each sample was run at the presented potential for 30 mins of electrolysis, after which the solution was tested immediately.

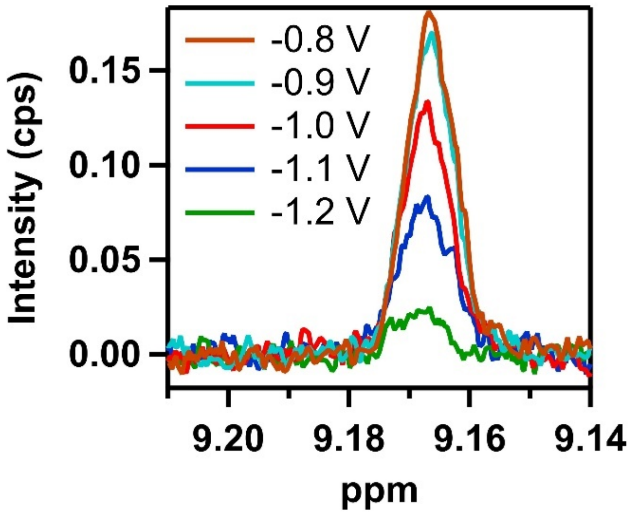

Figure S9: NMR spectra of the characteristic NAD^+ peak decreasing because of electrolysis at various potentials for 30 minutes. Each sample consisted of a 3.000 mL of 1.0 mM NAD^+.

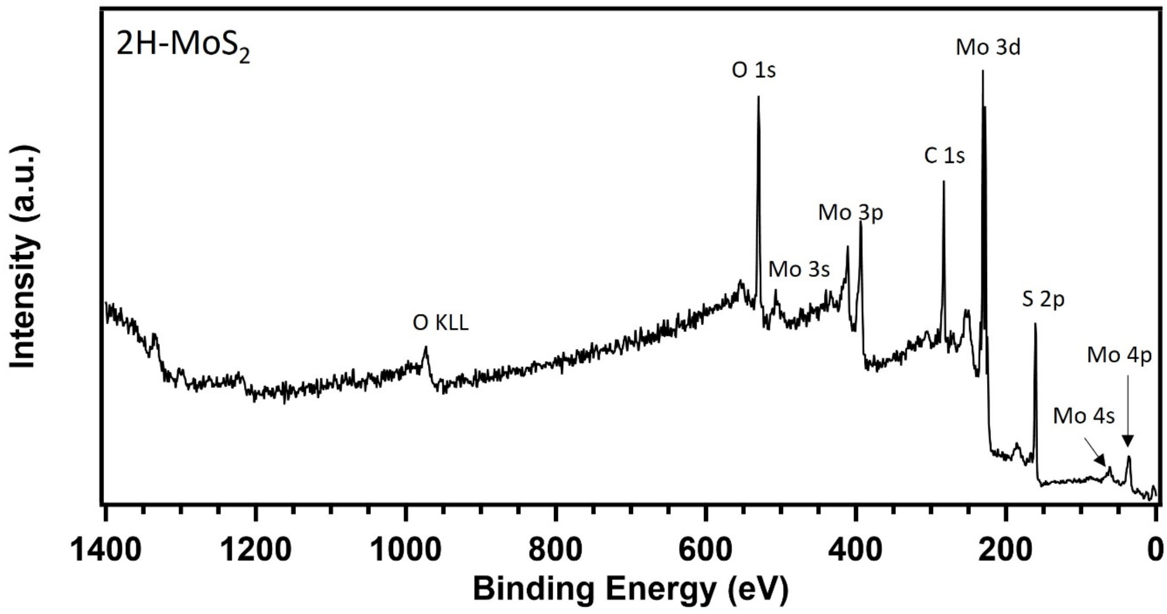

Figure S10: Survey Spectra 2H-MoS_2, which shows the presence of Mo, S, O, and C on the surface after annealing.

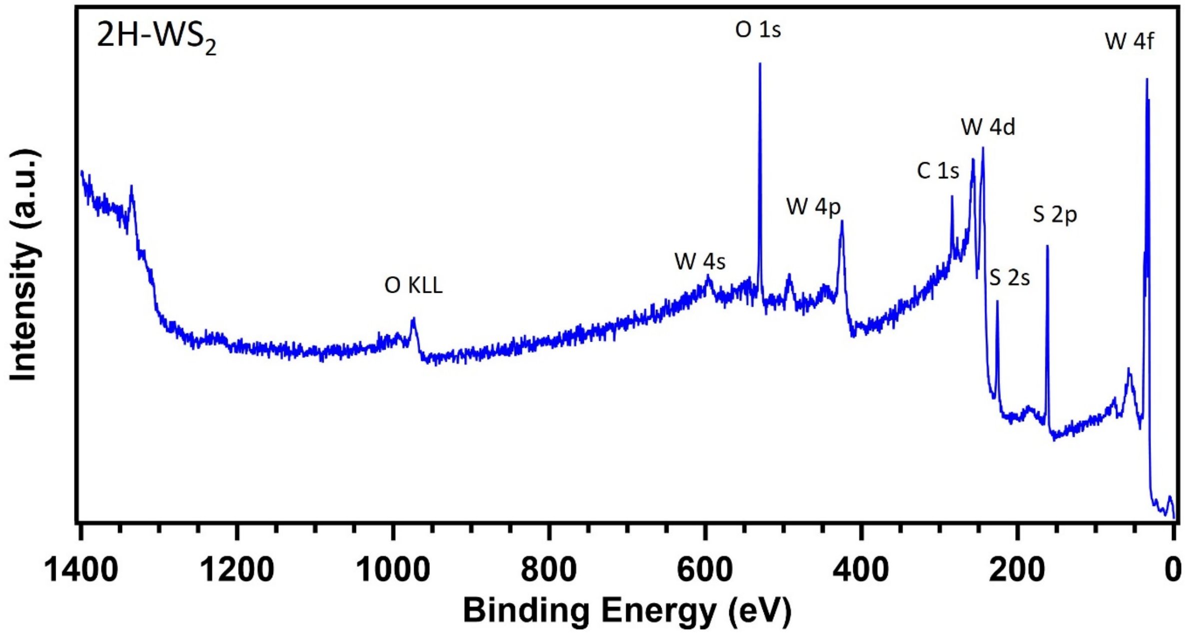

Figure S11: Survey Spectra 2H-WS_2, which shows the presence of W, S, O, and C are present on the surface after annealing.

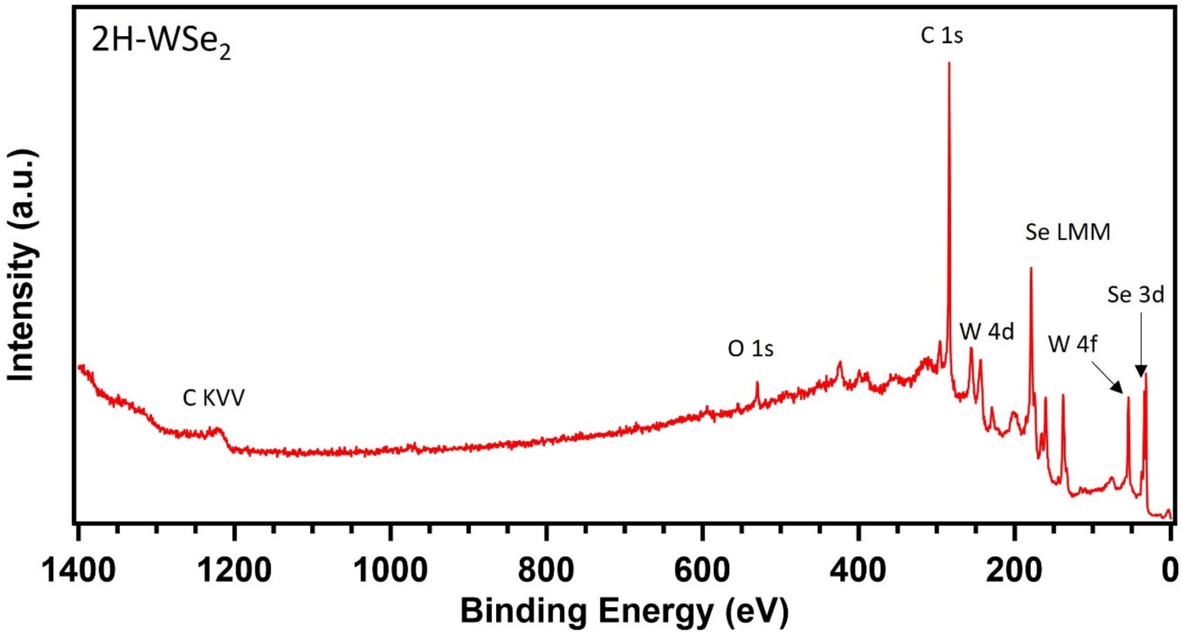

Figure S12: Survey Spectra 2H-WSe_2, which shows the presence of W, Se, O, and C are present on the surface after annealing**.**

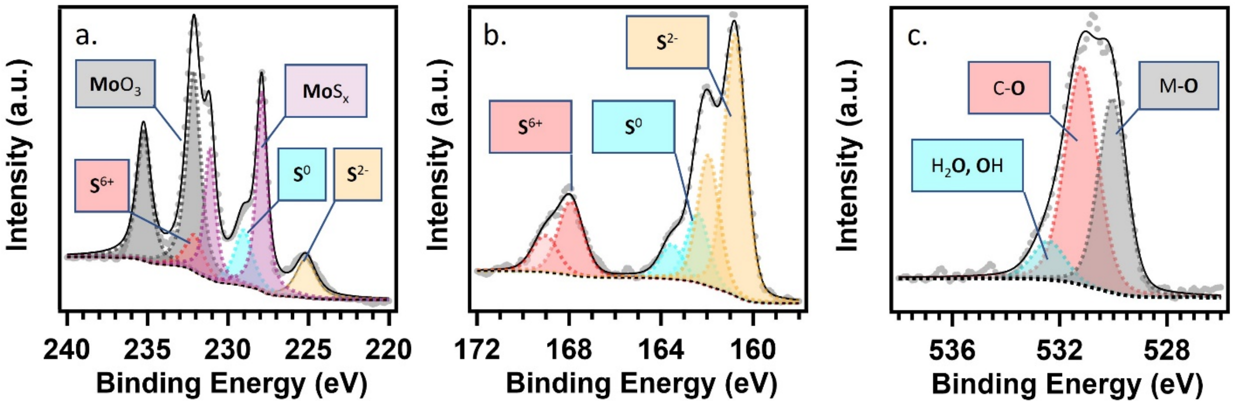

Figure S13: Detailed XPS spectra of MoS_x before annealing (a.) Mo 3d and S 2s region, (b.) S 2p region, and (c.) O 1s region.

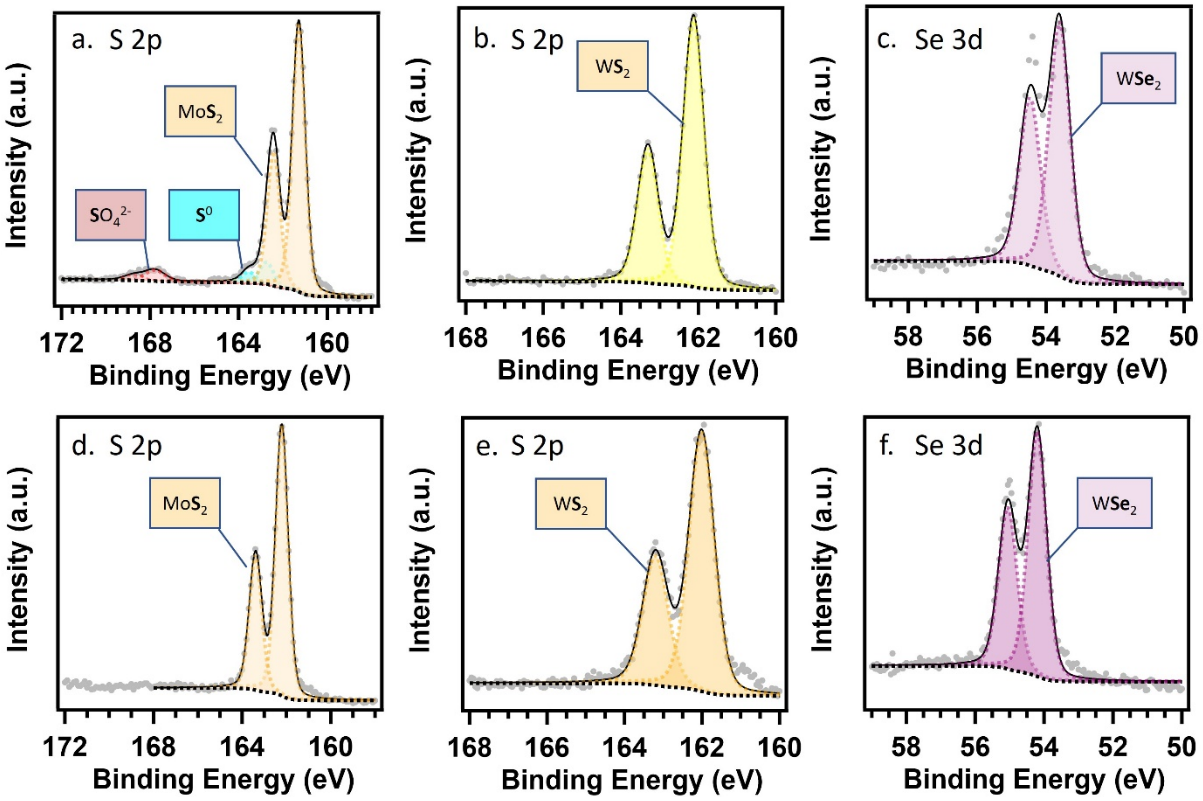

Figure S14: Detailed XPS spectra of 2H-MoS_2 collected before (a. b. and c.) and after electrolysis (d. e. f.) for 2H-MoS_2 (a, d), 2H-WS_2 (b, e), and 2H-WSe_2 (c, f). Electrolysis was conducted at -0.8 V vs. Ag/AgCl in an H-Cell with 0.1 M PBS electrolyte with N_2 sparging for 1200s.

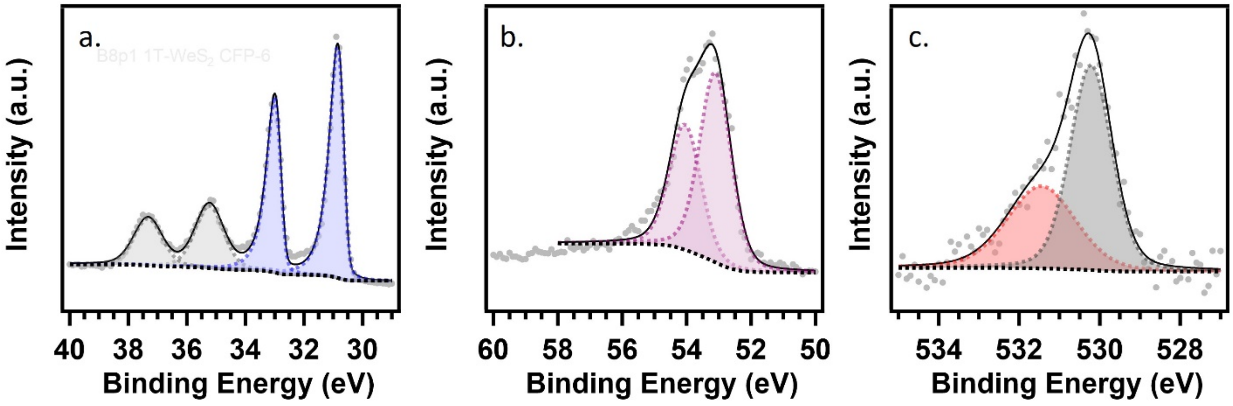

Figure S15: Detailed XPS spectra of 1T-WSe_2 (a.) W 3d region, (b.) Se 3d region, and (c.) O 1s region.

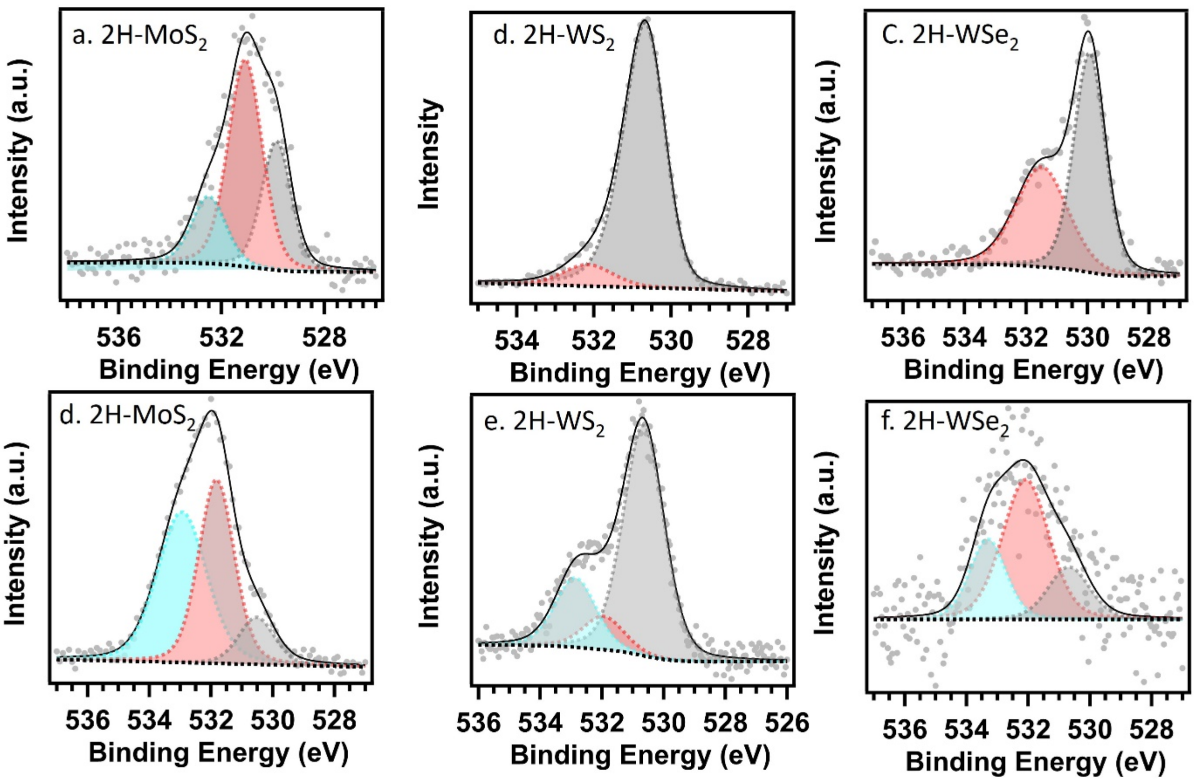

Figure S16: Detailed XPS spectra of O 1s core collected before (a. b. and c.) and after electrolysis (d. e. f.) for 2H-MoS_2 (a, d), 2H-WS_2 (b, e), and 2H-WSe_2 (c, f). Electrolysis was conducted at -0.8 V vs. Ag/AgCl in an H-Cell in 0.1 M PBS with N_2 sparging for 1200 s.

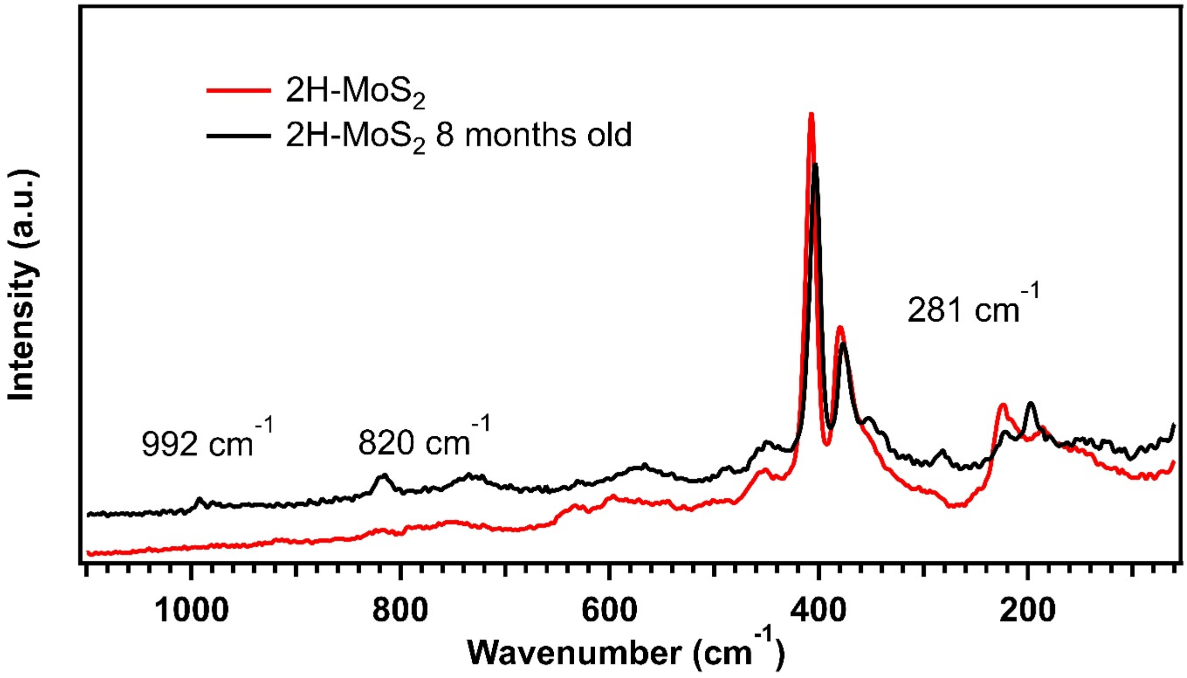

Figure S17: Raman spectra of 2H-MoS_2 substrates as fabricated and after eight months of storage under the ambient condition. MoO_3 with peak positions at 992 cm^{-1}, 820 cm^{-1}, and 281 cm^{-1} were identified.[1]

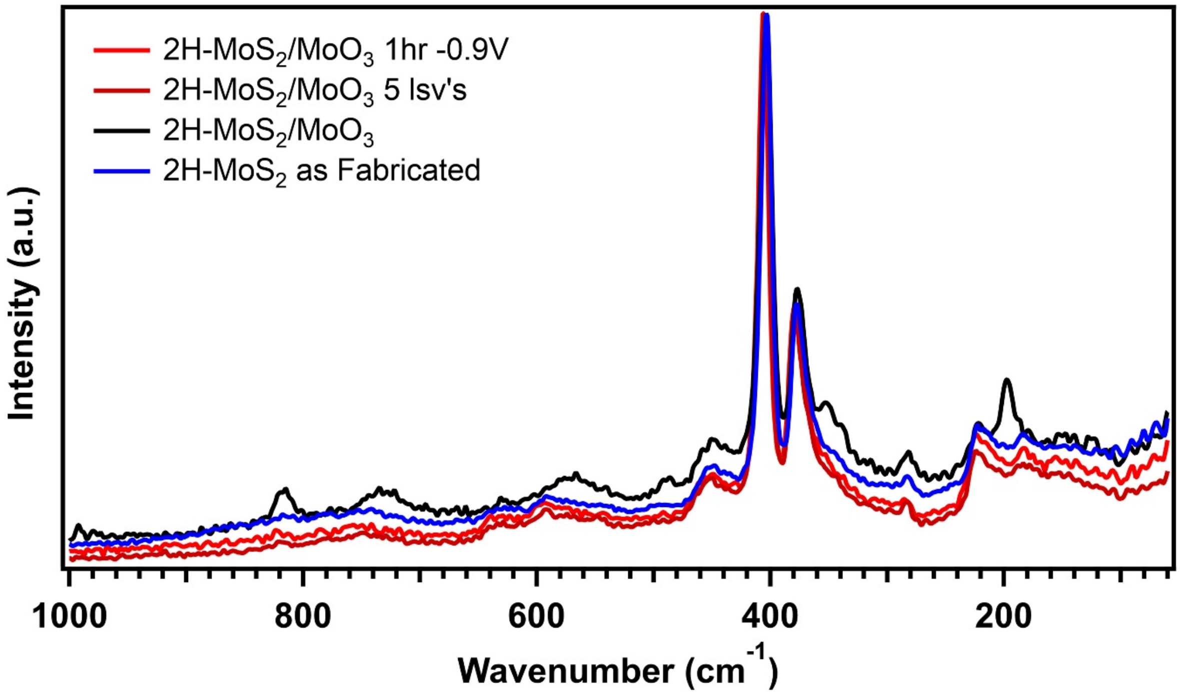

Figure S18: Raman spectra of $2H\text{-}MoS_2$ substrates as fabricated and after eight months of storage. The MoO_3 positioned at 992 cm^{-1}, 820 cm^{-1}, and 281 cm^{-1} were identified after eight months of storage. Linear sweeps voltammetry was conducted from 0 to -1.6 V vs. Ag/AgCl at 100 mV s^{-1}, and electrolysis was conducted at -0.9 V for one hour to remove the surface oxide layer. Ex-situ treatments were conducted in an H-Cell in N_2 sparged 0.1M PBS.

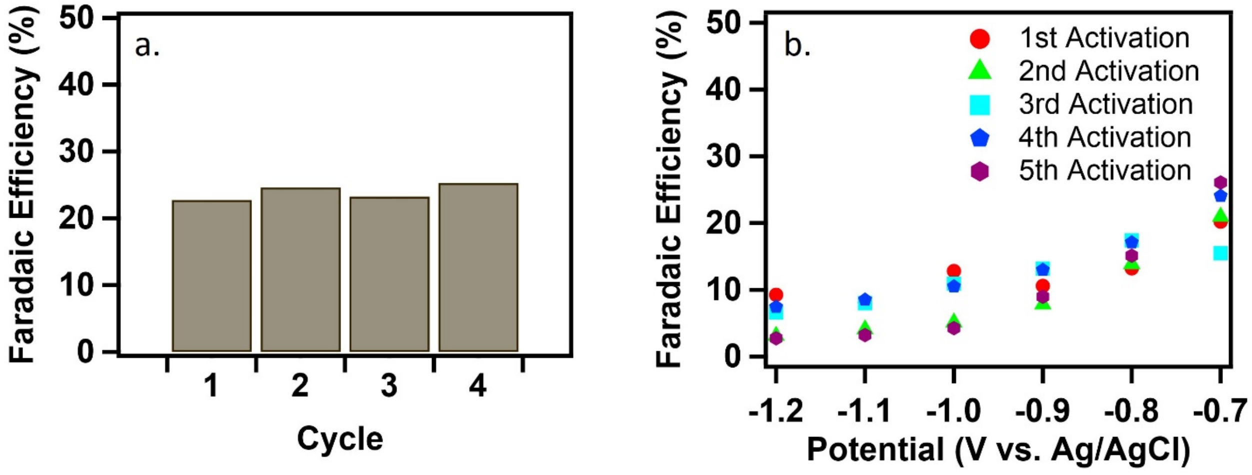

Figure S19: The stability of TMDCs, using (a.) 2H-MoS_2 and (b.) 2H-WS_2 electrodes, for electrochemical reduction of 1.0 mM NAD^+ (a.) FEs of 2H-MoS_2 at -0.8 V vs. Ag/AgCl for four subsequent NAD^+ experiments after electrode's initial activation and (b.) FEs of NADH from a single 2H-WS_2 electrode after five activation processes. Tests were conducted in an H-Cell with 0.1 M PBS electrolyte with N_2 protection.

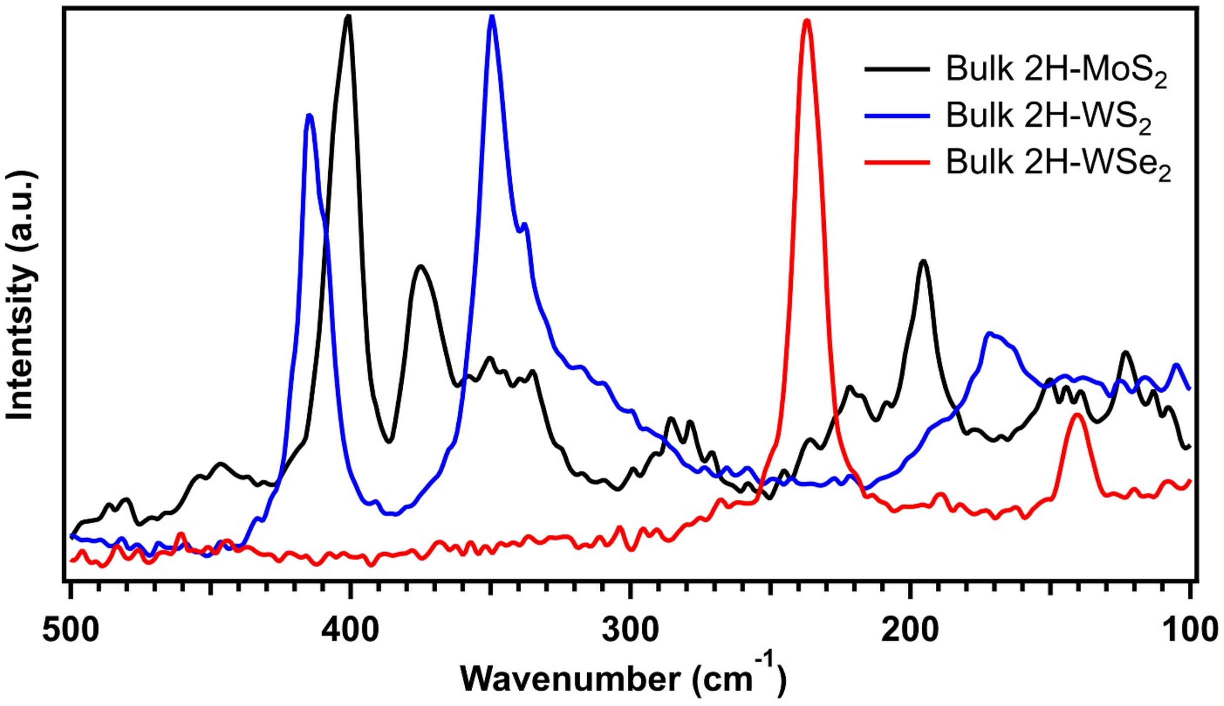

Figure S20: The Raman spectra of Bulk TMDCs collected after *in-situ* growth on CFP; products were washed with water and isolated through centrifugation.

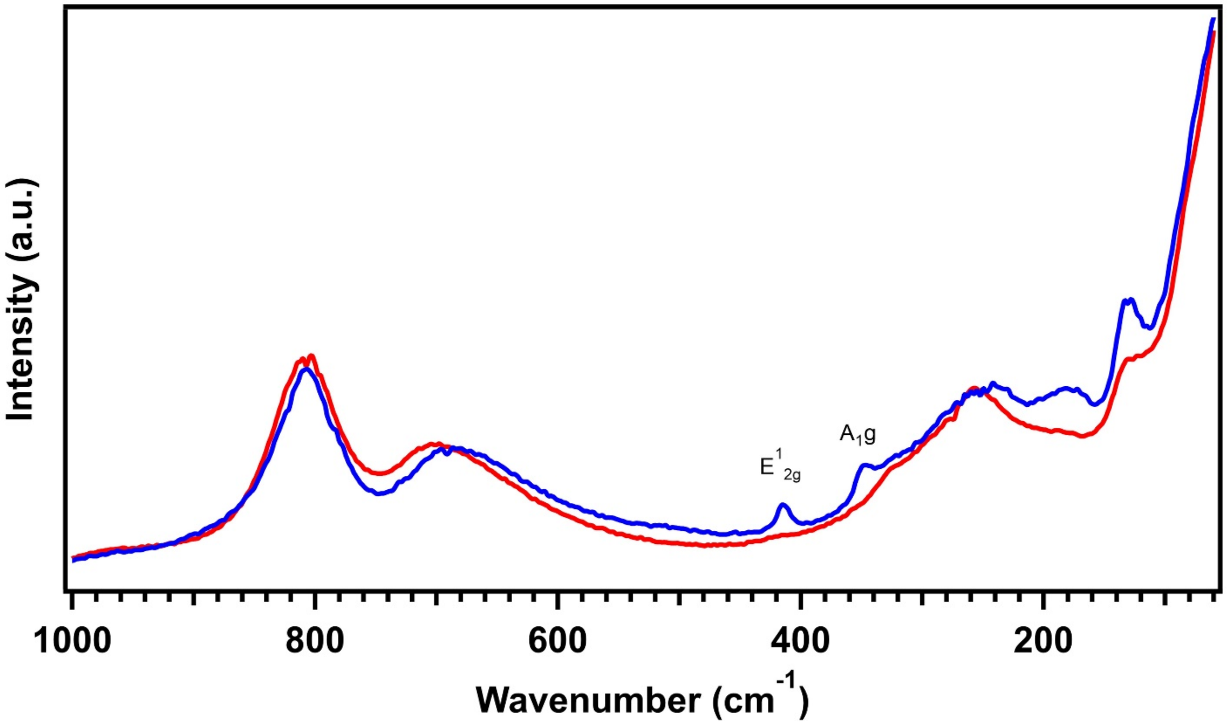

Figure S21: Raman spectra of bulk powder produced during WS_2 (blue) and WSe_2 (Red) fabrication without N_2 sparge. The presence of WO_3 is observed at 806 cm^{-1} and 686 cm^{-1}. Only A_{1g} and E^1_{2g} peaks of the 2H-WS_2 were observed and 2H-WSe_2 peaks were not observed in these spectra.

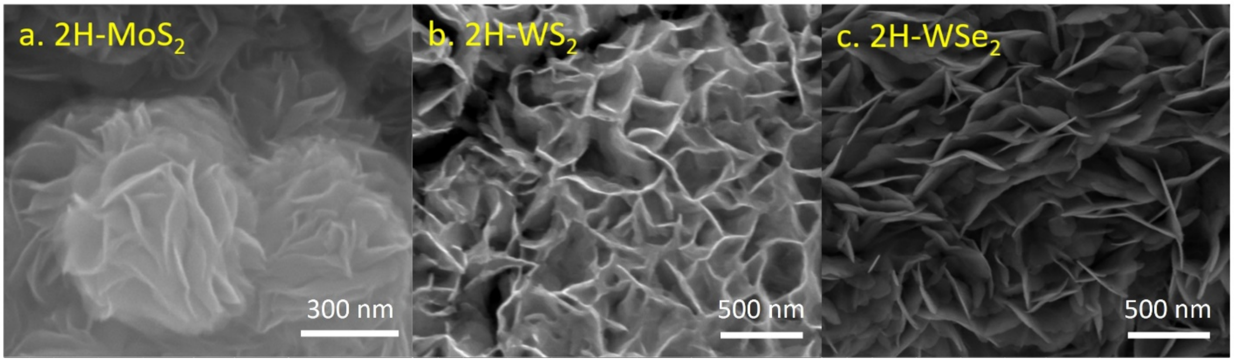

Figure S22: High magnification micrographs of (a.) 2H-MoS_2 (100 kX), b. 2H-WS_2 (50 kX), and c. 2H-WSe_2 (50 kX), were used to determine the widths of the nanoflower petals.

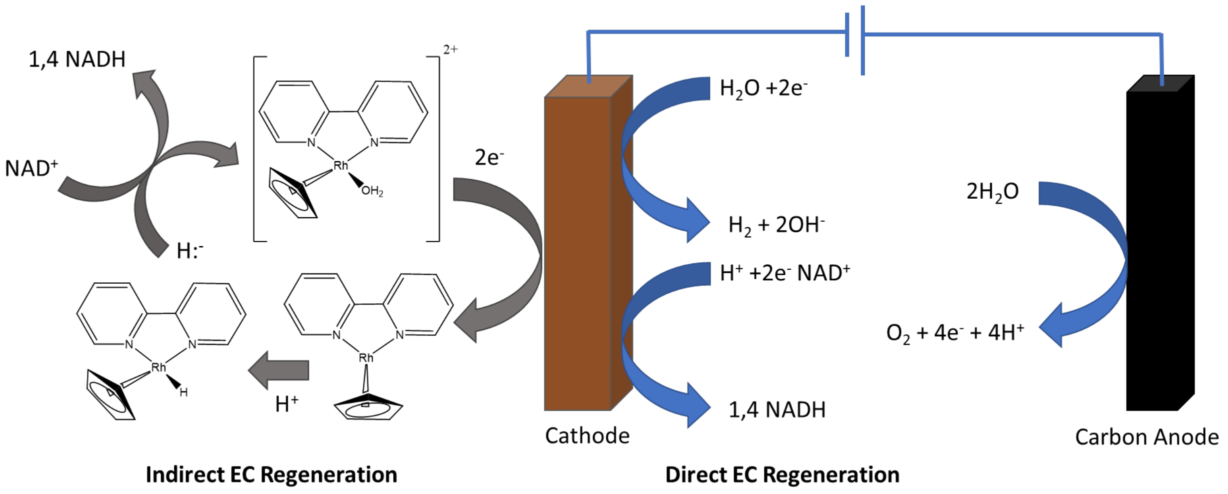

Scheme S1: Direct and indirect electrochemical regeneration of 1,4-NADH. Herein, 1,6-NADH was excluded from the direct EC regeneration.

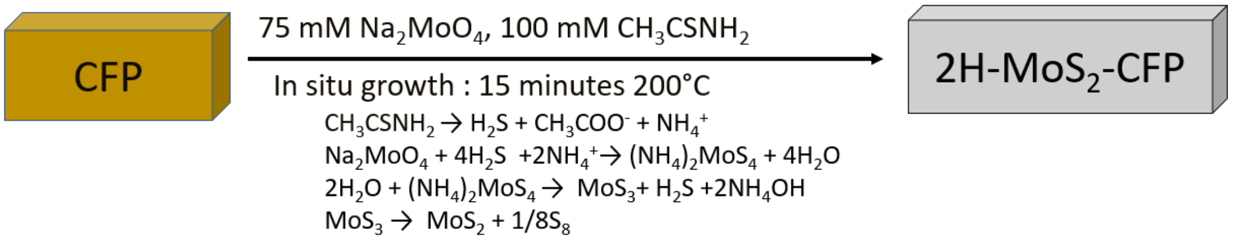

Supplementary scheme S2: MoS_2 microwave synthesis conditions and the possible reaction pathway.

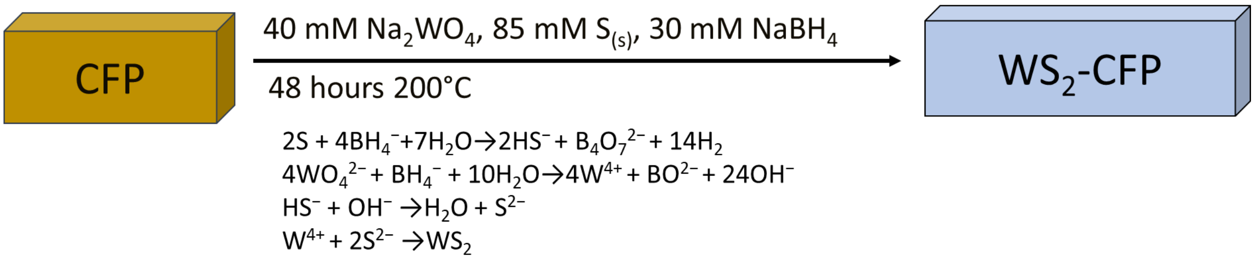

Supplementary scheme S3: WS_2 hydrothermal synthesis conditions and the possible reaction pathway.

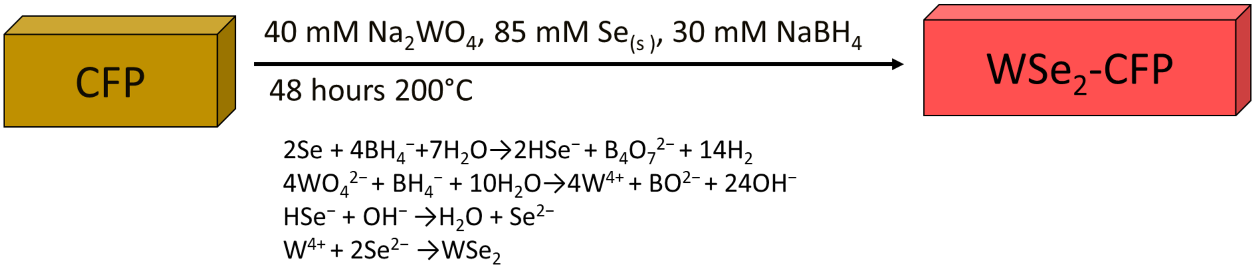

Supplementary scheme S4: WSe_2 hydrothermal synthesis conditions and the possible reaction pathway.

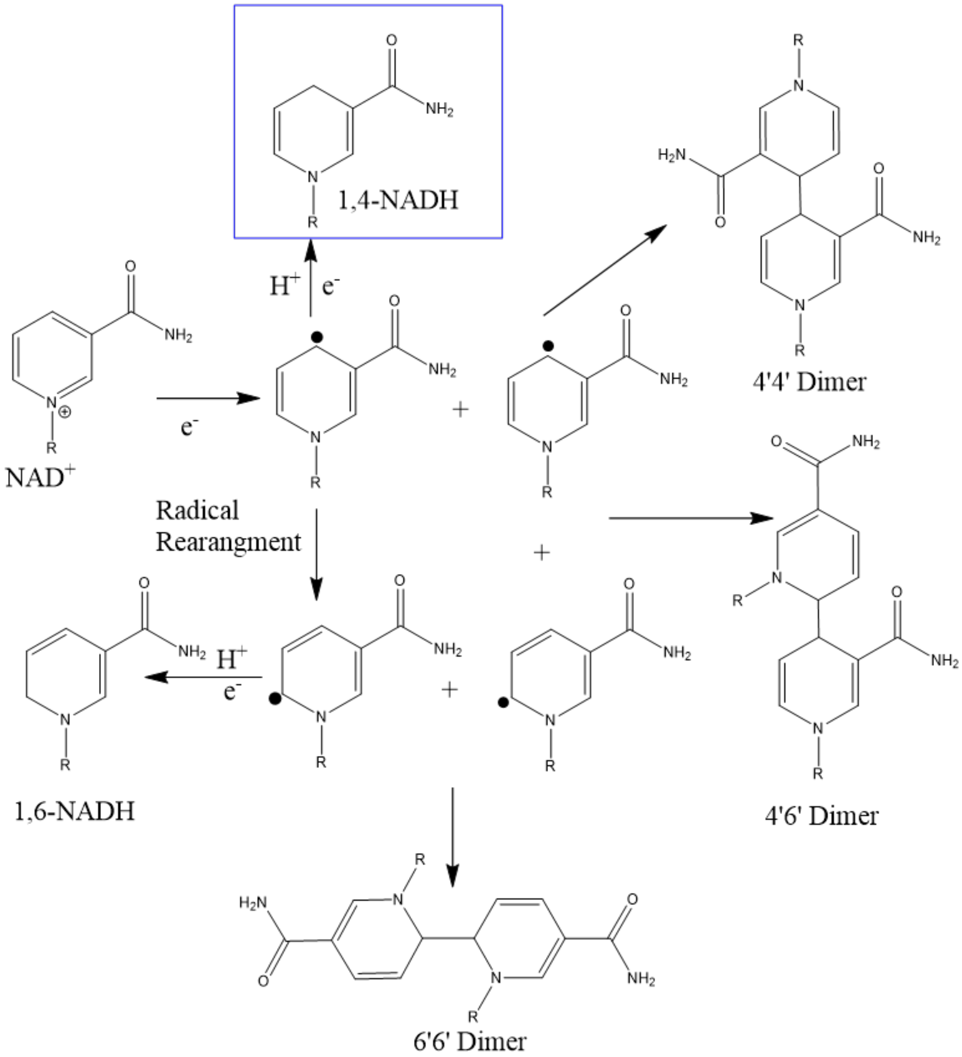

Scheme S5: Potential reaction pathways of NAD^+ electrochemical reduction and dimerization.

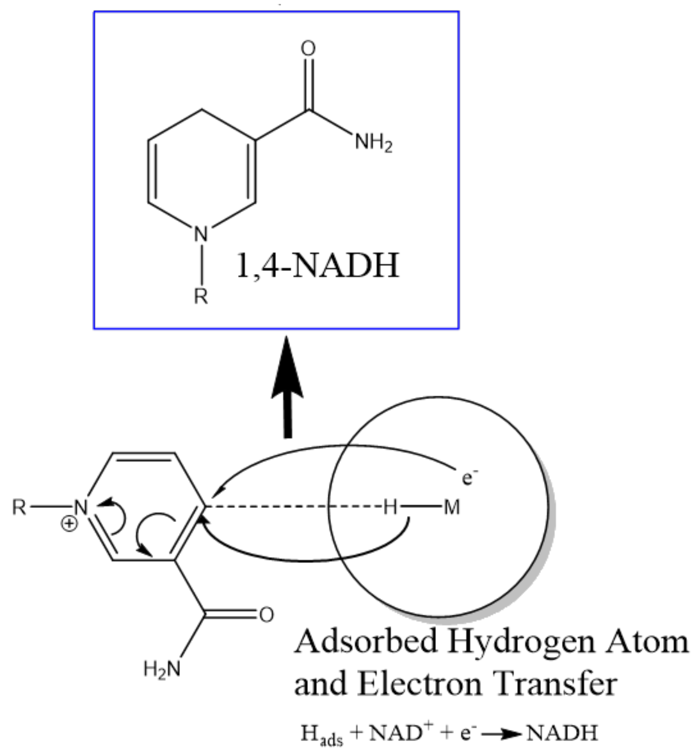

Scheme S6: Concerted NAD^+ reduction pathway for the formation of 1,4 NADH.

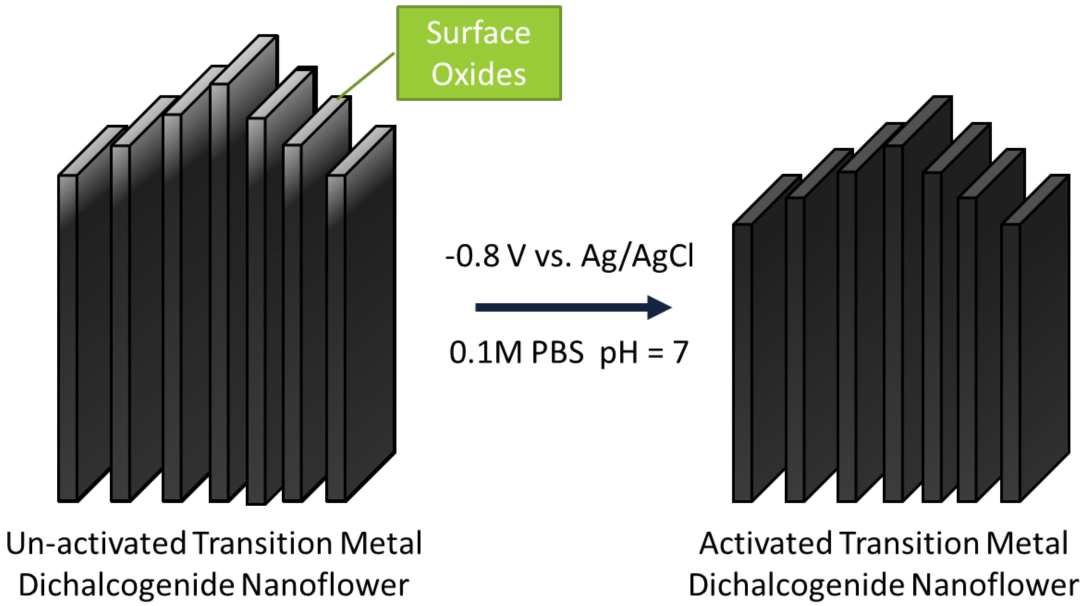

Scheme S7: Electrochemical oxide removal from the surface of TMDC nanoflowers.

Table S1: Recent results on electrochemical 1,4-NADH regeneration.

Electrode	Method	Potential Vs. Ag/AgCl	Selectivity	Faradaic Efficiency	Reference
GC	Direct EC	-1.82	100 %	n.a.	[2]
Pt-GC	Direct EC	-1.12	100%	n.a.	[2]
Ni-GC	Direct EC	-1.02	98%	n.a.	[2]
Ni	Direct EC	-1.50	92%	n.a.	[3]
Ti	Direct EC	-1.00	96%	n.a	[3]
Ni-NP-MWCNTS	Direct EC	-1.13	98%	n.a	[4]
[Rh(Cp∗)(bpy)Cl]	Direct EC	-0.73	Yes	87%	[5]
Immobilized diaphorase @ Cobaltocene functionalized electrode	Indirect	-0.86	100%	99%	[6]
Pt@GaAs	PEC	-0.75	50%	~15 %	[7]
Pt@GaAs	PEC	-0.95	80%	~5 %	[7]
**2H-WSe_2**	Direct EC	**-0.8**	**~100%**	**39 %**	**This work**
**2H-WS_2**	Direct EC	**-0.8**	**~91%**	**18 %**	**This work**
**2H-MoS_2**	Direct EC	**-0.8**	**~100%**	**19 %**	**This work**

Table S2: Raman peaks and peak assignments of 2H-phase TMDCs.

2H-WS_2		2H-MoS_2		2H-WSe_2	
Peak (cm^{-1})	Assignment	Peak (cm^{-1})	Assignment	Peak (cm^{-1})	Assignment
174.6	LA(M)	185.1	A_{1g} – LA(M)		
233.5	A_{1g}(M)-LA(M)	228.2	LA(M)		
349.2	2LA(M)	392	E^1_{2g}	246	A_{1g}/E_{2g}
354.5	E^1_{2g}	418	A_{1g}	303.39	B^1_{2g}
419.7	A_{1g}	455	2LA(M)		
521					
583.4	A_{1g}(M)+LA(M)				
692.9	4LA(M)				
1418	D-band Graphitic carbon				
1543	G-Band Graphitic carbon				
2864	2D-Band Graphitic carbon				

Table S3: Electrochemical overpotentials, j = 10 mA cm^{-2}, of TMDCs in different electrolytes and potentials (vs. RHE).

Sample	Nanoflower width (nm) SEM	Overpotential (0.5 M H_2SO_4)	Overpotential (0.1 M PBS)	C_{dl} (mF)
1T-WSe_2	16.46 ± 4.94	0.544	-	-
2H-WSe_2	18.56 ± 3.87	0.296	0.709	7.40
2H-WS_2	9.73 ± 3.46	0.248	0.781	13.50
2H-MoS_2	11.47 ± 1.95	0.325	0.676	2.55

Table S4: XPS assignments of the as synthesized and annealed TMDCs.

Assignment	1T-WSe_2 (eV)	2H-WSe_2 (eV)	MoS_x (eV)	2H-MoS_2 (eV)	2H-WS_2 (eV)
C-C, C=C C1s	284.8	284.8	284.8	284.8	284.8
C-O C1s		285.26	285.87	285.51	286.18
C=O C1s	286.89		288.3	286.86	288.14
(M-O) O1s	531.31	530.93	530.65	530.72	530.93
C-O O1s	532.54		531.51	531.95	531.79
H_2O; C=O O1s			532.31	533.61	532.91
Se^{2-} Se3d $_{5/2}$	54.2	54.87			
Se^{2-} Se3d $_{3/2}$	55.14	55.71			
S^{2-} S2p $_{3/2}$			161.61	162.34	161.78
S^{2-} S2p$_{1/2}$			162.76	163.47	162.97
S^{2-} S2s			225.99	226.68	226.18
W^{4+} W4f$_{7/2}$	31.98	32.66			32.13
W^{4+} W4f$_{5/2}$	34.13	34.79			34.28
W^{6+} W4f$_{7/2}$	36.32	35.94			35.46
W^{6+} W4f$_{5/2}$	38.41	38.23			37.71
Mo^{4+} 3d$_{5/2}$			228.72	229.52	
Mo^{4+} 3d$_{3/2}$			231.95	232.64	
Mo^{6+} 3d$_{5/2}$			232.96	233.39	
Mo^{6+} 3d$_{3/2}$			236.07	236.24	

Table S5: XPS binding energy separations of the fabricated and annealed TMDCs.

Assignment	1T-WSe_2 (eV)	2H-WSe_2 (eV)	MoS_x (eV)	2H-MoS_2 (eV)	2H-WS_2 (eV)
Se^{2-} $Se3d_{3/2}$ – Se^{2-} $Se3d_{5/2}$	0.94	0.84			
S^{2-} $S2p_{1/2}$ – S^{2-} $S2p_{3/2}$			1.15	1.13	1.19
W^{4+} $W4f_{5/2}$ – W^{4+} $W4f_{7/2}$	2.15	2.13			2.15
Mo^{4+} $3d_{3/2}$ – Mo^{4+} $3d_{5/2}$			3.23	3.12	
W^{6+} $W4f_{5/2}$ – W^{6+} $W4f_{7/2}$	2.09	2.29			2.25
Mo^{6+} $3d_{3/2}$ – Mo^{6+} $3d_{5/2}$			3.11	2.85	
W^{6+} $W4f_{7/2}$ – W^{4+} $W4\ f_{7/2}$	4.34	3.28			3.33
Mo^{6+} $3d_{3/2}$ – Mo^{4+} $3d_{5/2}$			7.35	6.72	

Table S6: Elemental ratios determined from the detail spectra where M represents metals and X represents chalcogenides.

	Electrolysis	M^{4+} : X^{2-}	M^{6+} : M-O O1s	M^{4+} : M^{6+}
1T-WSe_2	No	1:2.30	1 : 2.831	2.003 : 1
2H-WSe_2	No	1:1.90	1 : 2.850	4.100 : 1
2H-WSe_2	Yes	1:2.11	n/a	n/a
2H-WS_2	No	1:1.95	1 : 3.054	1.615 : 1
2H-WS_2	yes	1:1.88	1 : 3.46	3.775 : 1
MoS_x	No	1:2.22	1 : 2.526	0.785 : 1
2H-MoS_2	No	1:1.89	1 : 3.29	4.300 : 1
2H-MoS_2	Yes	1:1.97	n/a	n/a

Table S7: Binding energies separations of TMDCs after activation by 1200 s electrolysis at -0.8 V vs. Ag/AgCl in 0.1 M PBS.

Assignment	2H-WSe_2 (eV) Activated	2H-MoS_2 (eV) Activated	2H-WS_2 (eV) Activated
C-C, C=C C1s	284.8	284.8	284.8
C-O C1s	285.55	286.22	286.24
C=O C1s	286.7	288.41	288.08
(M-O) O1s	n/a	530.49	530.88
C-O O1s	531.97	531.78	532.18
H_2O; C=O O1s	533.46	532.92	533.09
Se^{2-} $Se3d_{5/2}$	54.8	n/a	n/a
Se^{2-} $Se3d_{3/2}$	55.69	n/a	n/a
S^{2-} $S2p_{3/2}$	n/a	162.18	162.24
S^{2-} $S2p_{1/2}$	n/a	163.36	163.43
S^{2-} S2s	n/a	226.41	226.58
W^{4+} $W4f_{7/2}$	32.75	n/a	32.63
W^{4+} $W4f_{5/2}$	34.89	n/a	34.77
W^{6+} $W4f_{7/2}$	n/a	n/a	35.77
W^{6+} $W4f_{5/2}$	n/a	n/a	38.00
W^{4+} $W4p_{3/2}$	37.49	n/a	n/a
Mo^{4+} $3d_{5/2}$	n/a	229.33	n/a
Mo^{4+} $3d_{3/2}$	n/a	232.5	n/a
Mo^{6+} $3d_{5/2}$	n/a	n/a	n/a
Mo^{6+} $3d_{3/2}$	n/a	n/a	n/a

Table S8: Binding energy separations of activated TMDCs after 1200s electrolysis at -0.8V vs. Ag/AgCl in 0.1 M PBS.

Assignment	2H-WSe_2 (eV) Activated	2H-MoS_2 (eV) Activated	2H-WS_2 (eV) Activated
Se^{2-} Se3d $_{3/2}$ - Se^{2-} Se3d $_{5/2}$	0.89		
S^{2-} S2p$_{1/2}$ - S^{2-} S2p $_{3/2}$		1.18	1.19
W^{4+} W4f$_{5/2}$ – W^{4+} W4f$_{7/2}$	2.14		2.14
Mo^{4+} 3d$_{3/2}$- Mo^{4+} 3d$_{5/2}$		3.17	
W^{6+} W4f$_{5/2}$ - W^{6+} W4f$_{7/2}$			2.23
W^{6+} W4f$_{7/2}$ – W^{4+} W4f$_{7/2}$			3.14

References:

1. Windom, B. C.; Sawyer, W. G.; Hahn, D. W., A Raman Spectroscopic Study of MoS_2 and MoO_3: Applications to Tribological Systems. *Tribol. Lett.* **2011,** *42* (3), 301-310.
2. Ali, I.; Gill, A.; Omanovic, S., Direct Electrochemical Regeneration of the Enzymatic Cofactor 1,4-NADH Employing Nano-patterned Glassy Carbon/Pt and Glassy Carbon/Ni Electrodes. *Chem. Eng. J.* **2012,** *188*, 173-180.
3. Ali, I.; Khan, T.; Omanovic, S., Direct Electrochemical Regeneration of the Cofactor NADH on Bare Ti, Ni, Co and Cd Electrodes: The Influence of Electrode Potential and Electrode Material. *J. Mol. Catal. A: Chem.* **2014,** *387*, 86-91.
4. Ali, I.; Ullah, N.; McArthur, M. A.; Coulombe, S.; Omanovic, S., Direct Electrochemical Regeneration of Enzymatic Cofactor 1,4-NADH on a Cathode Composed of Multi-walled Carbon Nanotubes Decorated with Nickel Nanoparticles. *Can. J. Chem. Eng.* **2017,** *96* (August 2017), 68-73.
5. Zhang, L.; Vilà, N.; Kohring, G. W.; Walcarius, A.; Etienne, M., Covalent Immobilization of (2,2′-Bipyridyl) (Pentamethylcyclopentadienyl)-Rhodium Complex on a Porous Carbon Electrode for Efficient Electrocatalytic NADH Regeneration. *ACS Catal.* **2017,** *7* (7), 4386-4394.
6. Yuan, M.; Kummer, M. J.; Milton, R. D.; Quah, T.; Minteer, S. D.; Shelley, D., Efficient NADH Regeneration by a Redox Polymer-Immobilized Enzymatic System. *ACS Catal.* **2019**, 5486-5495.
7. Stufano, P.; Paris, A. R.; Bocarsly, A., Photoelectrochemical NADH Regeneration using Pt-Modified p-GaAs Semiconductor Electrodes. *ChemElectroChem* **2017,** *4* (5), 1066-1073.
